# Pathways of Iron and Sulfur Acquisition, Cofactor Assembly, Destination, and Storage in Diverse Archaeal Methanogens and Alkanotrophs

**DOI:** 10.1128/JB.00117-21

**Published:** 2021-08-09

**Authors:** Christina Johnson, Alexis England, Mason Munro-Ehrlich, Daniel R. Colman, Jennifer L. DuBois, Eric S. Boyd

**Affiliations:** a Department of Chemistry and Biochemistry, Montana State University, Bozeman, Montana, USA; b Department of Microbiology and Immunology, Montana State University, Bozeman, Montana, USA; University of Illinois at Urbana Champaign

**Keywords:** *Archaea*, iron uptake, methanogens, sulfur

## Abstract

Archaeal methanogens, methanotrophs, and alkanotrophs have a high demand for iron (Fe) and sulfur (S); however, little is known of how they acquire, traffic, deploy, and store these elements. Here, we examined the distribution of homologs of proteins mediating key steps in Fe/S metabolism in model microorganisms, including iron(II) sensing/uptake (FeoAB), sulfide extraction from cysteine (SufS), and the biosynthesis of iron-sulfur [Fe-S] clusters (SufBCDE), siroheme (Pch2 dehydrogenase), protoheme (AhbABCD), cytochrome *c* (Cyt *c*) (CcmCF), and iron storage/detoxification (Bfr, FtrA, and IssA), among 326 publicly available, complete or metagenome-assembled genomes of archaeal methanogens/methanotrophs/alkanotrophs. The results indicate several prevalent but nonuniversal features, including FeoB, SufBC, and the biosynthetic apparatus for the basic tetrapyrrole scaffold, as well as its siroheme (and F_430_) derivatives. However, several early-diverging genomes lacked SufS and pathways to synthesize and deploy heme. Genomes encoding complete versus incomplete heme biosynthetic pathways exhibited equivalent prevalences of [Fe-S] cluster binding proteins, suggesting an expansion of catalytic capabilities rather than substitution of heme for [Fe-S] in the former group. Several strains with heme binding proteins lacked heme biosynthesis capabilities, while other strains with siroheme biosynthesis capability lacked homologs of known siroheme binding proteins, indicating heme auxotrophy and unknown siroheme biochemistry, respectively. While ferritin proteins involved in ferric oxide storage were widespread, those involved in storing Fe as thioferrate were unevenly distributed. Collectively, the results suggest that differences in the mechanisms of Fe and S acquisition, deployment, and storage have accompanied the diversification of methanogens/methanotrophs/alkanotrophs, possibly in response to differential availability of these elements as these organisms evolved.

**IMPORTANCE** Archaeal methanogens, methanotrophs, and alkanotrophs, argued to be among the most ancient forms of life, have a high demand for iron (Fe) and sulfur (S) for cofactor biosynthesis, among other uses. Here, using comparative bioinformatic approaches applied to 326 genomes, we show that major differences in Fe/S acquisition, trafficking, deployment, and storage exist in this group. Variation in these characters was generally congruent with the phylogenetic placement of these genomes, indicating that variation in Fe/S usage and deployment has contributed to the diversification and ecology of these organisms. However, incongruency was observed among the distribution of cofactor biosynthesis pathways and known protein destinations for those cofactors, suggesting auxotrophy or yet-to-be-discovered pathways for cofactor biosynthesis.

## INTRODUCTION

Iron (Fe) and sulfur (S) are required by nearly all forms of life, where they are essential constituents of the biocatalytic machines that power cells. These include simple and complex iron-sulfur ([Fe-S]) cluster and siroheme or heme cofactors in enzymes and proteins that are critical for cellular function and that contribute collectively to global redox chemistry and element cycles (1). Examples of widespread and biogeochemically important enzymes and proteins whose functions depend on [Fe-S] clusters include simple electron transfer ferredoxins (Fds), as well as more complex multielectron redox metalloenzymes that catalyze reversible hydrogen oxidation (hydrogenases), dinitrogen reduction (nitrogenases), and oxidation of water (photosystem I) (2, 3). Likewise, iron protoporphyrin IX and its derivatives (collectively, heme cofactors) are components of a variety of gas transporters, electron transfer proteins (cytochromes), sensor regulators, and enzymes that catalyze diverse redox reactions (4–6), while the related cofactor siroheme is involved in sulfite and nitrite reduction (7). Collectively, these cofactors leverage iron to move electrons in bond-making/-breaking or energy-harvesting processes.

Methanogens are a diverse and ancient (8–11) group of organisms that have been classified as belonging to more deeply diverging type I (*Methanopyrales*, *Methanobacteriales*, and *Methanococcales*) or more recently diverging type II (*Methanocellales*, *Methanomicrobiales*, and *Methanosarcinales*) lineages (8, 10, 12). Type I methanogens have been suggested to have diversified >3.9 billion years ago (Ga) (9, 11, 13), whereas members of the type II lineage have been estimated to have diverged as recently as 3.1 Ga (13). Since this classification was initially introduced, several sublineages of the *Methanosarcinales* have been discovered that catalyze anaerobic methane oxidation, as reviewed in Knittel and Boetius (14). Furthermore, several putative archaeal methanogen, methanotroph, and alkanotroph lineages that do not fall within this previously defined classification scheme, including members of the *Archaeoglobales*, *Bathyarchaeota*, *Crenarchaeota*, *Hadesarchaeota*, *Verstraetearchaeota*, and *Methanomassiliicoccales* (15–17), have recently been discovered. Collectively, these studies have greatly expanded the taxonomic, functional, and ecological diversity of archaeal methanogens, methanotrophs, and alkanotrophs and have changed our understanding of their evolution (18).

Several key differences have been noted in the use of Fe- and S-dependent electron carriers and the source of S for biosynthesis in prior studies of representative methanogens from type I and type II lineages, reflecting differences in environmental element availability, their evolutionary history, or both. First, it has been noted that methanogens, on the whole, appear to utilize [Fe-S] clusters to a greater extent than other facultative anaerobic or aerobic cells, such as Escherichia coli (19, 20). The greater reliance on [Fe-S] clusters in methanogens has been suggested to result from increased bioavailability of Fe and S in anoxic early Earth environments, when the primary diversification of methanogens is thought to have taken place (21). However, once oxygenic *Cyanobacteria* capable of oxidizing water to generate oxygen (O_2_) evolved, near-surface environments became more oxidized, resulting in a lower inventory of bioavailable Fe and S (22). These studies imply that the type and use of electron carriers in biology has been closely tied to the availability of Fe (and possibly S) through Earth’s history. Second, cofactor preference appears to differ along taxonomic lines, with the use of heme-containing cytochromes potentially restricted to type II methanogen cells. Furthermore, the source of S for use in [Fe-S] cluster biosynthesis appears to be different, with type I cells apparently more reliant on sulfide as the primary S donor and type II cells being reliant on or at least capable of using cysteine as a sulfur donor (20, 21). This is consistent with previous informatics analyses of the sulfur uptake pathway for [Fe-S] biosynthesis in methanogens (20, 23), which identified a nearly ubiquitous distribution of homologs of cysteine desulfurase that liberate persulfide from cysteine (24) in type II cells but only rarely identified them in type I cells. It is unclear how other recently discovered archaeal methanogen and alkanotroph phyla (i.e., *Bathyarchaeota*, *Crenarchaeota*, *Hadesarchaeota*, and *Verstraetearchaeota*) acquire and deploy Fe and S and whether an understanding of these metabolic capabilities might provide new insight into their relationships with the better understood species of types I and II. Given broad similarities in the metabolism and ecology (e.g., strict anaerobes) of archaeal methanogens, methanotrophs, and alkanotrophs (25, 26), it is possible that these newly described organisms share similarities in cofactor usage and Fe/S metabolic pathways with type I or II cells. However, very little is known of cofactor usage or Fe/S metabolic pathways across the diversity of described archaeal methanogens, methanotrophs, and alkanotrophs. This is largely attributed to only a few species being studied in the laboratory and the number of organisms that are only known to exist via metagenomic sequencing of environmental DNA and subsequent genome reconstruction.

In the present study, a total of 326 genomes were compiled from cultivars and metagenome-assembled genomes (MAGs) corresponding to members of *Archaea* either demonstrated or inferred to be methanogens, methanotrophs, or alkanotrophs based on metabolic reconstructions (see Data Set S1 in the supplemental material). All genomes encoded homologs of methyl coenzyme M reductase (MCR), a genetic marker for methanogenesis, methanotrophy, and alkanotrophy among *Archaea* (26, 27). These genomes and MAGs were screened for homologs of proteins that have been shown, in at least some archaeal and/or bacterial species, to be involved in the acquisition and/or storage of Fe and S and for homologs of proteins involved in [Fe-S] cluster and/or (siro)heme assimilation and biosynthesis. Finally, to assess the extent of cofactor usage, genomes were screened for homologs of proteins or for protein motifs that bind a variety of [Fe-S] clusters, heme *b*, heme *c*, and siroheme. The distribution of these homologs and motifs was examined within a phylogenomic framework, with the goal of identifying unique physiological features of each of the groups of methanogens, methanotrophs, and alkanotrophs that can inform on their ecology and evolution.

## RESULTS AND DISCUSSION

### Motivation for and overview of bioinformatics approach.

Archaeal methanogens (11), methanotrophs (28), and alkanotrophs (26) are ancient groups of microorganisms that, in the case of the former, are known to have a high demand for Fe and S (19, 20). Methanococcus maripaludis (type I) cells, for example, were shown to contain ∼15-fold more [Fe-S] clusters per milligram of protein than E. coli cells (20), and the genomes of several model methanogens code for a higher number of [Fe-S] cluster binding motifs than those of facultative and obligate aerobes (19). This suggests a trend toward greater dependence of type I methanogen cells on [Fe-S] clusters for electron transfer and catalysis, which we examined more extensively here. In particular, little is known of the relative abundances of [Fe-S] cluster binding proteins and/or motifs in deeply diverging type I and type II methanogens or among the large number of recently discovered archaeal methanogens, methanotrophs, and alkanotrophs (15–17) that do not clearly fall into one of these previously established groups (29). Furthermore, a strict delineation has been proposed for the use of cytochromes among type II cells but not type I cells (30), based on the relatively small number of available genomes from model species at that time.

**FIG 1:**
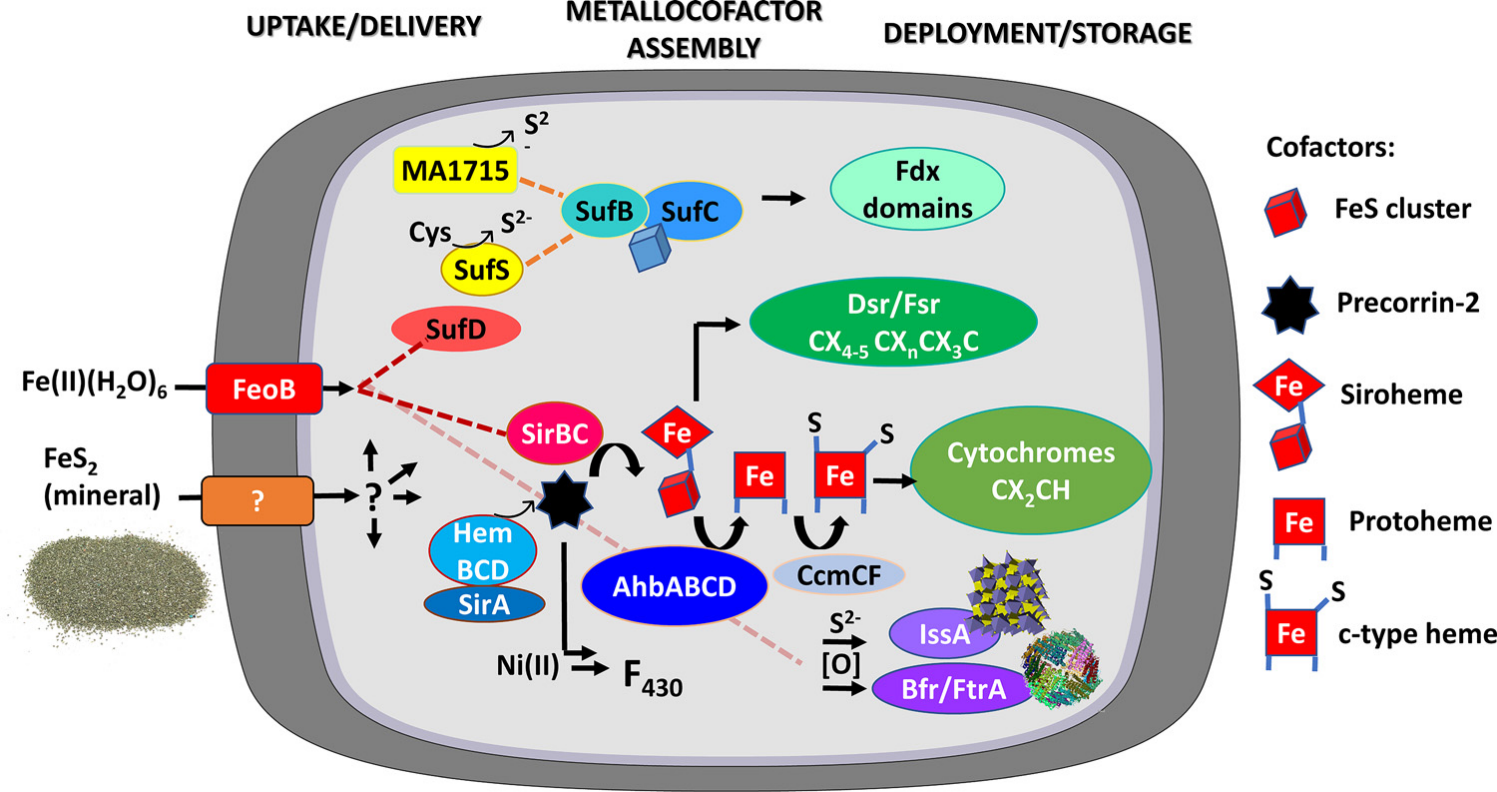
Schematic illustrating putative pathways involved in the uptake and processing of iron and sulfur and their putative cellular destinations in archaeal methanogens, methanotrophs, and alkanotrophs. Proteins involved in iron (Fe) uptake (FeoB) or trafficking (SufD) are in shades of red; those involved in sulfur (S) extraction from cysteine (SufS) are in yellow; those involved in iron-sulfur ([Fe-S]) cluster (SufBCDE), siroheme (SirABC), protoheme (AhbABCD), and cytochrome *c* (CcmCF) cofactor biosynthesis are in blue; those involved in cofactor binding/use (Fdx domains, Dsr, Fsr, and cytochromes) are in green; and those involved in S and/or Fe storage/detoxification (IssA, Bfr, and FtrA) are in purple. The shapes of the symbols reflect the types of cofactors that are being synthesized/trafficked, as defined in the key on the right. Question marks depict yet-to-be-identified proteins or pathways. Enzyme names are as follows: FeoB, ferrous iron transporter; SufS, cysteine desulfurase; SufBCDE, sulfur pathway for [Fe-S] biosynthesis; SirABC, precorrin-2 dehydrogenase; AhbABCD, alternative heme biosynthesis proteins; HemBCD, tetrapyrrole biosynthesis proteins; CcmCF, cytochrome *c* biosynthesis proteins; Dsr, F_420_-independent dissimilatory sulfite reductase; Fsr, F_420_-dependent sulfite reductase; IssA, iron sulfur storage protein; Bfr, bacterioferritin; FtnA, ferritin; Fdx, ferredoxin. Example [Fe-S] cluster binding motifs (CX_4-5_CX*_n_*CX_3_C) and heme (CX_2_CH) binding motifs are depicted. Chemical abbreviations are as follows: FeS_2_, pyrite or iron disulfide; Fe(II)(H_2_O)_6_, hexaquo Fe(II); Cys, cysteine; S^2−^, sulfide; Fdx, ferredoxin; O, oxygen.

It remains to be determined how [Fe-S] and heme cofactor usage is partitioned among the genomes of diverse methanogen, methanotroph, and alkanotroph cells, many of them newly sequenced, and how cofactor usage reflects the evolution of these species. These gaps in understanding motivated the informatics analyses conducted herein to examine the distribution of homologs of proteins and pathways putatively involved in the acquisition, trafficking, and processing of Fe and S in archaeal methanogen, methanotroph, and alkanotroph genomes and MAGs. The following sections are laid out sequentially to first examine the evolutionary history of archaeal methanogens and alkanotrophs using phylogenomic approaches. Next, proteins and pathways inferred to be involved in the acquisition, trafficking, and processing of Fe and S were compiled using informatics approaches. Then, the potential destinations for (siro)heme and [Fe-S] clusters among proteomes inferred from genomic data are presented. For organizational purposes, each section begins with an overview of each enzyme or enzyme pathway, followed by a cross-comparison of what was found through informatics approaches. A schematic depicting target proteins and pathways that were examined is provided in Fig. 1 (see also Data Set S2 for searched protein sequences). Additionally, we searched for cofactor-binding proteins and sequence motifs, which are reported in Data Sets S3 and S4 and in Materials and Methods.

### Phylogenomic analysis of archaeal methanogens and alkanotrophs.

Previous phylogenetic analyses have assigned methanogens either to the deeply diverging type I lineage (*Methanopyrales*, *Methanobacteriales*, and *Methanococcales*) or the more recently diverging type II lineage (*Methanocellales*, *Methanomicrobiales*, and *Methanosarcinales*) (8, 10, 12). However, several recent cultivation and (meta)genomic-sequencing efforts have expanded the diversity of known methanogens, including the discovery of members of the *Methanomassiliicoccales* (17), which can be found in habitats as diverse as the gut microbiomes of humans, termites, and millipedes, as well as non-host-associated rice paddies, wetlands, and subseafloor and freshwater sediments (31), in addition to hypersaline environments (Methanonatronarchaeum thermophilum), as demonstrated by the newly proposed methanogen order, *Methanonatronarchaeales* (32). Furthermore, putative archaeal methanogens, methanotrophs, and alkanotrophs, as inferred through metabolic predictions (e.g., genomes that encode methyl coenzyme M reductase [MCR], including members of the *Archaeoglobales*, *Bathyarchaeota*, *Crenarchaeota*, *Hadesarchaeota*, and *Verstraetearchaeota* [15–17, 33]), do not clearly fall out among previously classified type I and II lineages (29). Bathyarchaeotes encoding MCR have been identified in environments as diverse as terrestrial aquifers (16) and hydrothermal systems (34). Members of the *Archaeoglobales*, *Crenarchaeota*, *Hadesarchaeota*, and *Verstraetearchaeota* that encode MCR also have a wide ecological distribution, with MAGs reported from marine and terrestrial hydrothermal systems (33–35) and anaerobic digestors (15).

**FIG 2:**
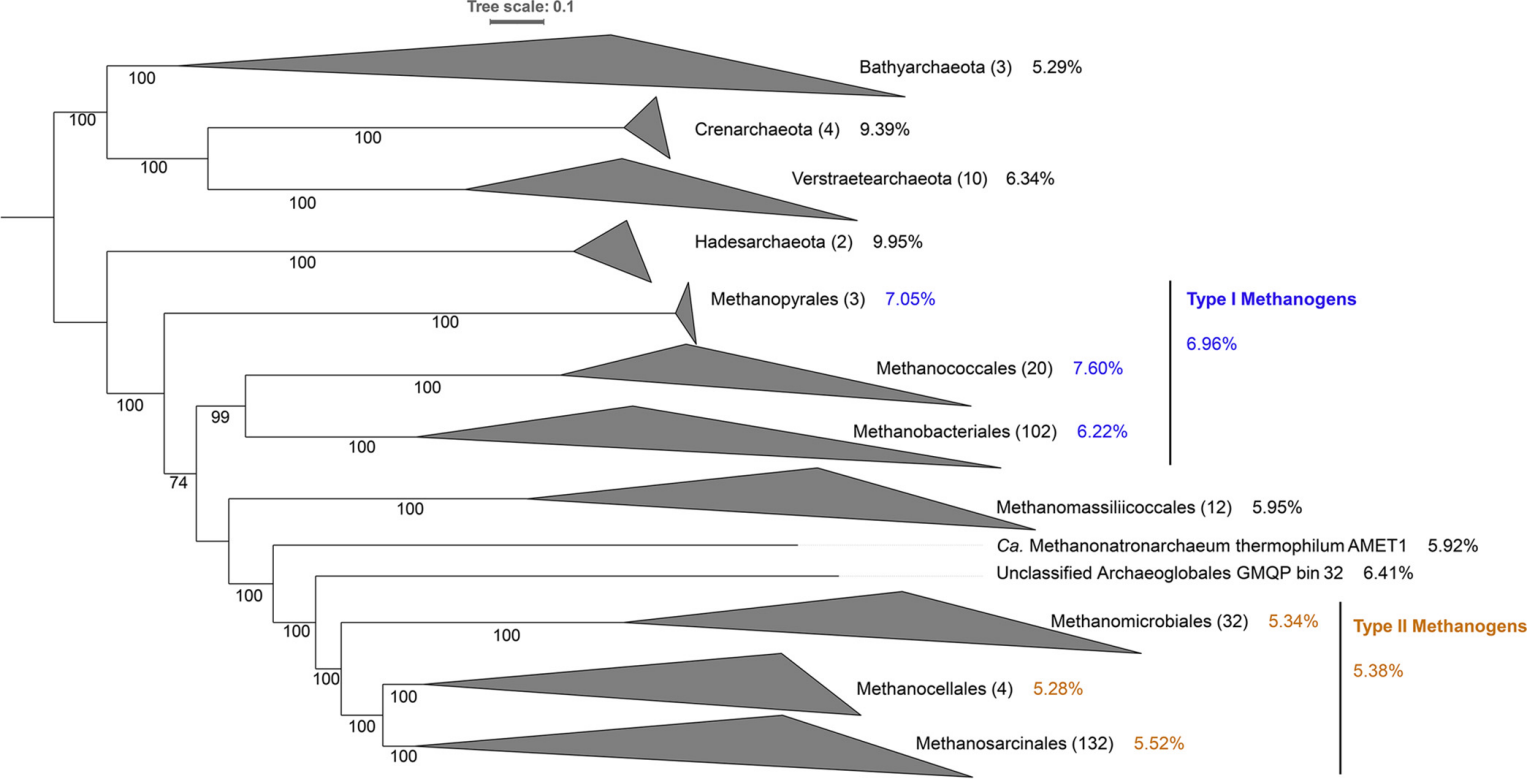
Maximum-likelihood phylogenomic analysis of 326 archaeal methanogen, methanotroph, and alkanotroph genomes or metagenome-assembled genomes (MAGs) analyzed as part of this study. Phylogenomic reconstruction of the archaeal genomes or MAGs was conducted by surveying them for 103 archaeon-specific single-copy housekeeping phylogenetic-marker genes. Individual protein coding genes were compiled, aligned, and concatenated. The concatenated alignment was then subjected to maximum-likelihood phylogenetic analysis. Lineages are collapsed at the order or phylum taxonomic rank, and lineages corresponding to type I and type II methanogens are indicated by blue and orange colors, respectively, and by line depictions. Percentages shown next to taxonomic assignments reflect the average percentage of proteins encoded in those specified genomes that contain [Fe-S] binding motifs. Numbers at nodes show bootstrap support (out of 1,000 replicates), and the scale at the top shows the expected number of substitutions per site in the alignment. The numbers in parentheses next to each clade are the number of genomes or MAGs included in each of those clades. Importantly, only putative archaeal methanogens, methanotrophs, and alkanotrophs were included in this phylogeny. The phylogenetic positions of archaeal methanogens, methanotrophs, and alkanotrophs in reference to 2,337 genomes or MAGs reflecting the current known breadth of archaeal diversity are presented in Fig. S1.

To provide an evolutionary framework that includes these new lineages of archaeal methanogens, methanotrophs, and alkanotrophs for use in evaluating the distribution of proteins and pathways involved in Fe and S acquisition, trafficking, and processing and to determine the destinations of (siro)heme and [Fe-S] clusters, a maximum-likelihood phylogenomic reconstruction of the 326 genomes and MAGs was performed (Fig. 2). Importantly, the phylogenetic positions of methanogens, methanotrophs, and alkanotrophs relative to other archaeal lineages can be found in Fig. S1. Phylogenetic reconstruction of the 326 genomes and MAGs revealed two primary lineages, one of which included members of the *Bathyarchaeota*, *Crenarchaeota*, and *Verstraetearchaeota* and comprised putative methanogens, methanotrophs, and alkanotrophs. Genomes affiliated with *Bathyarchaeota* branched sister to a lineage comprising the genomes of *Crenarchaeota* and *Verstraetearchaeota*. The second primary lineage included genomes affiliated with the *Hadesarchaeota*, the type I and II methanogens/methanotrophs, methanogenic members of the *Methanomassiliicoccales*, and the single genome of the methanogen Methanonatronarchaeum thermophilum. Genomes affiliated with the *Hadesarchaeota* formed the base of the second lineage, with genomes/MAGs representing the *Methanomassiliicoccales* and M. thermophilum forming lineages that branch between the type I and type II methanogen/methanotroph lineages. This tree topology is consistent with those generated recently that include representatives of these novel lineages (18). Moreover, the branching topology shown here is consistent with an *Archaea*-wide phylogenomic analysis incorporating 2,337 representative genomes from across all known major archaeal groups (Fig. S1). These observations point to the need to characterize Fe and S usage, in particular as it relates to [Fe-S] and heme cofactors, in deeply diverging *Bathyarchaeota*, *Crenarchaeota*, *Hadesarchaeota*, and *Verstraetearchaeota*, as well as in more recently evolved *Methanomassiliicoccales* and M. thermophilum, to see if patterns emerge that can be used to infer differences in cofactor usage among these taxa.

### Iron(II) import.

Fe is the most abundant transition metal on Earth, and its ability to cycle between oxidized and reduced forms has led to its ubiquitous role as an electron carrier and cofactor component (36). Differences in the chemical speciation and bioavailability of iron under oxic versus anoxic conditions have likely led to the diversification of cellular mechanisms for iron acquisition, incorporation, and packaging into protein-associated cofactors. While Fe is water soluble in its reduced form [Fe(II)], when oxidized and at circumneutral pH, it forms ferric oxyhydroxide compounds that have low solubility (37). Informatics analyses reported herein are focused on known mechanisms for cellular import of Fe(II), since this is a likely source of Fe in anoxic environments inhabited by archaeal methanogens, methanotrophs, and alkanotrophs. Importantly, several methanogens and methanotrophs have been shown to reduce Fe(III) oxides (e.g., see references 38 and 39). It is possible that Fe(II) that is generated through dissimilatory Fe(III) reduction could be transported into the cell; however, evidence in support of this functionality has yet to be reported.

FeoB is a G protein-coupled channel protein that serves as a major point of entry for hexaquo Fe(II) {[Fe(II)H_2_O]_6_} and, to a lesser extent, Mn(II) across the (inner) membrane (40) of diverse *Bacteria* (41). The transmembrane FeoB protein is generally accompanied by a soluble component (FeoA) that serves a regulatory function (41). Nucleoside triphosphate (NTP)-driven, FeoAB-mediated transport of Fe(II) has also been suggested to be the primary mechanism of acquiring this metal in methanogens (42). Homologs of FeoAB were identified in every order and in 91% and 77% of the surveyed type I and type II genomes/MAGs herein, respectively (Table 1). Among unclassified MAGs, 36% encoded homologs of FeoAB; however, since many of these MAGs are incomplete (Data Sets S1 and S3), it is possible that these organisms also use FeoAB to sense and import Fe(II).

**TABLE 1:** Distribution of protein homologs involved in iron and sulfide assimilation and [Fe-S] cluster biosynthesis among 326 archaeal methanogen, methanotroph, and alkanotroph genomes

Taxonomic group	Total no. of genomes^*a*^	No. of complete genomes/no. of draft genomes	No. of genomes with protein homologs involved in:							% of proteins with [Fe-S] motifs^*c*^
			Fe(II)(H_2_O)_6_ uptake			[Fe-S] biosynthesis				
			FeoAB	FeoA only	FeoB only	SufBC^*b*^	SufS	SufD	SufE	
Type I										
* Methanobacteriales*	102	21/81	96	0	5	96	95	0	1	6.22
* Methanococcales*	20	15/5	15	0	5	20	6	0	0	7.60
* Methanopyrales*	3	3/0	3	0	0	3	2	0	0	7.05

Type II										
* Methanocellales*	4	3/1	1	0	3	4	4	0	0	5.28
* Methanomicrobiales*	32	11/21	16	0	15	30	30	0	0	5.34
* Methanosarcinales*	132	37/95	112	0	21	127	127	0	10	5.52

Unclassified										
* Archaeoglobales*	1	0/1	0	0	1	1	0	0	0	6.41
* Bathyarchaeota*	3	0/3	0	0	1	2	3	0	0	5.29
* Crenarchaeota*	4	0/4	1	0	2	2	2	0	0	9.39
* Hadesarchaeota*	2	0/2	0	0	1	2	2	0	0	9.95
* Methanomassiliicoccales*	12	4/8	8	1	1	10	9	1	0	5.95
* Methanonatronarchaeales*	1	0/1	0	0	0	1	1	0	0	5.92
* Verstraetearchaeota*	10	0/10	3	0	4	9	8	0	0	6.34

a The number of genomes within a defined taxonomic group that encoded one or more homologs is indicated. Searched sequences (Data Set S2), the distribution of homologs and their accession numbers among the 326 individual genomes (Data Set S3), and information about the searched genomes (Data Set S1) are provided in the supplemental material.

b Genomes were only counted positive if homologs of both SufBC were encoded in the genome.

c The average percentage of proteins in the genomes of members of each specified taxonomic group with putative [Fe-S] cluster binding domains is shown.

Fifty-nine genomes (18% of the total) of type I or II methanogens/methanotrophs encoded homologs of only FeoB without the accompanying regulatory subunit FeoA. Of these, 38 were draft genomes, suggesting that the absence of FeoA may be attributed to incomplete sequencing. However, 21 of the genomes encoding only FeoB were complete, including 4 type I *Methanococcales*, 11 type II *Methanosarcinales*, 4 type II *Methanomicrobiales*, and 2 type II *Methanocellales* genomes (Data Set S3). Lack of FeoA is consistent with its nonessential role among *Bacteria* (41). Furthermore, the use of FeoB is apparently not universal in archaeal methanogens, methanotrophs, and alkanotrophs, as three reportedly complete genomes (Methanolacinia petrolearia DSM 11571, “*Candidatus* Methanoplasma termitum” Mpt1, and Methanothrix thermoacetophila PT) and another 17 draft genomes did not possess a homolog.

The absence of FeoA in many methanogens/methanotrophs/alkanotrophs suggests the possibility of alternative mechanisms for sensing Fe and regulating FeoB-mediated Fe uptake, while the lack of FeoB implies the existence of yet-to-be-defined pathways for acquiring iron in these cells. Novel uptake mechanisms or regulatory pathways could include still-unidentified but FeoB-like NTP-dependent transporters or, potentially, proteins attuned to the chemical species of Fe and S available in anoxic environments inhabited by these organisms. Recently, for example, the uptake of Fe(II) in the form of iron-sulfur mineral species was described for Methanococcus voltae A3 (type I) and Methanosarcina barkeri MS (type II) (43). Iron-sulfur minerals like iron disulfide (pyrite; FeS_2_) and iron monosulfide (mackinawite; FeS) (44) and soluble [Fe-S] aqueous clusters (45) are common in sulfidic and anoxic environments, including those typically inhabited by these organisms (21). Cultures of M. voltae and M. barkeri provided with FeS_2_ catalyzed the reductive dissolution of the mineral, leading to the generation of sulfide (HS^−^) and iron monosulfide, the latter of which is thought to be assimilated to meet Fe and S demands (43). While the genes necessary for FeS_2_ reduction and iron monosulfide assimilation are not yet known, it is possible that they are widespread among methanogens, methanotrophs, and alkanotrophs, considering that representatives of both type I and type II cells exhibited this phenotype. Furthermore, given broad similarities in the metabolism and ecology (e.g., anaerobes) of archaeal methanogens, methanotrophs, and alkanotrophs (25), it is possible that archaeal methanotrophs and alkanotrophs can meet Fe (and S) demand using iron sulfides.

### Distribution of pathways for sulfur uptake and assimilation into [Fe-S] clusters.

Bioinformatic (e.g., see references 19 and 46) and whole-cell (e.g., see references 20, 47, and 48) studies have shown that protein-associated [Fe-S] clusters constitute a major destination for both Fe and S in methanogens, and the same is expected of archaeal methanotrophs and alkanotrophs, given the broad similarities in their core metabolisms (49). We therefore considered sulfur assimilation in the context of how [Fe-S] cluster biosynthesis pathways are sourced with sulfur. Three pathways for [Fe-S] cluster biosynthesis are known (reviewed in reference 2), including the iron sulfur cluster (ISC), the sulfur (SUF), and the nitrogen fixation (NIF) pathways (50). The ISC pathway is common among *Bacteria*, the SUF pathway is common in both *Bacteria* and *Archaea*, and the NIF pathway is restricted to a subset of bacterial organisms that encode molybdenum nitrogenase (2). First described in E. coli (50), the full SUF system is comprised of genes *sufABCDSE* (51, 52), though many microbial cells, in particular members of *Archaea*, only encode a subset (20, 23). In E. coli, the SUF system is suspected to function during periods of iron starvation or oxidative stress, while the ISC pathway functions under typical growth conditions (53). A recent informatics study showed that *Archaea* only encode SUF, and it is therefore presumed to be the primary [Fe-S] assembly system in archaeal cells (23). Although SUF has not been extensively studied in any archaeon, the core complex in E. coli is made up of the [Fe-S] scaffold proteins SufB and SufD and an ATPase, SufC (50). Phylogenetic and informatics studies suggest that SufB is ancestral to SufD, with the latter arising from a duplication of an ancestor of *sufB* (23). Homologs of both SufBC were identified in 311 of the 326 (95%) genomes of type I and II and unclassified putative methanogens, methanotrophs, and alkanotrophs (Table 1); 14 genomes that did not encode a homolog of SufB or SufC were incomplete and often encoded other Suf homologs (Data Set S3). Despite being complete, the genome of “*Ca.* Methanoplasma termitum” Mpt1 (*Methanomassiliicoccales*), a hydrogen-dependent methylotrophic methanogen isolated from a termite gut (54), did not encode homologs of SufBC but did encode a homolog of SufS, indicating that it can obtain S from cysteine (Data Set S3). However, the apparent absence of SufBC raises the intriguing question of whether these cells synthesize [Fe-S] clusters or if they possibly can directly assimilate [Fe-S], as has recently been proposed for several type I and II methanogens (43). Together, these observations suggest that the vast majority of the archaeal methanogens, methanotrophs, and alkanotrophs surveyed herein utilize the core Suf system (SufBC) for [Fe-S] cluster biosynthesis.

Cysteine, the substrate of cysteine desulfurase, is a primary source of S for [Fe-S] cluster synthesis in bacterial and eukaryotic pathways. Homologs of cysteine desulfurase (SufS) were identified in 290 of the 311 (93% of total) genomes that encoded both SufB and SufC (Table 1). Importantly, 9 draft and 12 complete genomes encoded SufBC but not a homolog of SufS. The 12 complete genomes where a homolog of SufS was not identified were from the order *Methanococcales*, including several *Methanococcus* and *Methanocaldococcus* species. This is consistent with previous informatics studies that did not detect a homolog of SufS or other cysteine desulfurases in the genomes of several methanogens and other nonmethanogenic *Archaea* (20, 23). Follow-on laboratory experiments with M. maripaludis S2, whose genome does not encode a homolog of SufS, revealed that sulfide is the sulfur source for this strain (20). This indicates the presence of a yet-to-be-defined mechanism for liberating S from cysteine for [Fe-S] cluster biosynthesis in M. maripaludis. More recent studies have suggested that the addition of sulfide to medium containing trace elements including Fe(II) initiates the formation of iron mono- and disulfides, both of which can be used to meet the Fe and S demands of type I and type II methanogens (43). This may suggest that the acquisition of SufS in type I and, ultimately, type II methanogen, methanotroph, and alkanotroph cells resulted in an expanded spectrum of the types of S sources that can support cellular demands. The detection of SufS in many newly identified archaeal methanogens, methanotrophs, and alkanotrophs, including members of the *Bathyarchaeota*, *Crenarchaeota*, *Hadesarchaeota*, *Methanomassiliicoccales*, *Methanonatronarchaeales*, and *Verstraetearchaeota*, suggests this to be a mechanism by which these cells can obtain S for [Fe-S] cluster biosynthesis as well.

Homologs of SufD, which derives from a duplication of an ancestor of *sufB*, were nearly universally absent from genomes (325/326 genomes) (Table 1), consistent with the suggestion that this duplication took place after the divergence and specialization of methanogens (23). Like SufD, homologs of the sulfur carrier protein SufE were rarely identified among the genomes surveyed, with the majority of homologs detected among recently evolved *Methanosarcinales* (Table 1). Previous studies have suggested a possible connection between SufD and an iron acquisition step during [Fe-S] cluster assembly in E. coli (55). Furthermore, biochemical and genetic experiments have shown a role for SufE as a S carrier during [Fe-S] cluster assembly (56). Based on a previous analysis of the taxonomic and phylogenetic distribution of SufD and SufE across archaeal and bacterial genomes, they were hypothesized to have been recruited to the *suf* operon to allow a more controlled delivery of Fe(II) and S, respectively, to the scaffold SufBC (23). Furthermore, recruitment of these genes to the *suf* operon was suggested to be a possible response to an increase in O_2_ availability (23). The rarity of SufD and SufE homologs in the genomes surveyed here, all of which are likely to be strict anaerobes, is consistent with this suggestion.

It is possible that methanogens, particularly those lacking SufS, make direct use of some form of sulfide (S^2−^). HS^−^ is the dominant form of reduced, soluble sulfur in anoxic environments, including those in which methanogens reside (21), and a likely source of their nutritional sulfur. Many type I and type II methanogens/methanotrophs require HS^−^ for growth, and yet, high concentrations of HS^−^ can be toxic to cells (57, 58). Sulfide readily forms precipitates with transition metal ions, including Fe(II), nickel [Ni(I) and (II)], zinc [Zn(II)], and copper [Cu(I)], yielding aqueous metal-sulfide clusters (45, 59) that can structurally transform into more stable mineral structures (44) and relieve toxicity (60). While HS^−^ in a nonionic state (H_2_S) has been proposed to passively diffuse across the cell membrane, where it is met by enzymes involved in trafficking and assimilation into [Fe-S] clusters or other organic sulfur-containing compounds (61, 62), the observation of iron-sulfur mineral uptake (43) suggests undiscovered pathways of accessing sulfur directly from mineralized metal sulfides. These pathways would likewise act to minimize intracellular HS^−^ concentrations (43).

### Distribution of [Fe-S] cluster binding motifs.

Previous studies noted that M. maripaludis S2 cells use a greater number of [Fe-S] clusters than E. coli (20) and encode a greater number of [Fe-S] binding motifs in their inferred proteomes than facultative anaerobes and aerobes (19). Furthermore, a recent informatics/phylogenetics study suggests that more deeply diverging organisms utilize [4Fe-4S] Fds to a greater extent than those that are more recently evolved (46). Here, we examined the distribution of homologs of 50,339 proteins that have verified [Fe-S] cluster binding motifs among the 326 archaeal methanogen, methanotroph, and alkanotroph genomes using hidden Markov model (HMM) profiles that were developed for [Fe-S] binding motifs or domains, as defined in the Protein Family (Pfam) database and as implemented with the program MetalPredator (63). These data were then used to calculate the percentage of assembled proteins in each of these genomes that are predicted to bind one or more [Fe-S] clusters (Data Set S3). This percentage value was then averaged for all genomes within each taxonomic order (Table 1) and mapped on the phylogenomic tree (Fig. 2) to look for broader phylogenetic patterns in the data.

Roughly 5.29 and 6.34% of the proteins encoded in the genomes of *Bathyarchaeota* and *Verstraetearchaeota* members, which comprise two lineages of archaeal methanogens/methanotrophs/alkanotrophs, were predicted to bind [Fe-S] clusters (Table 1 and Fig. 2). Within this group, the genomes of *Crenarchaeota* (9.39%) are more enriched in [Fe-S] binding proteins than are those of the *Bathyarchaeota* and *Verstraetearchaeota*. Similarly, within the second primary stem lineage (Fig. 2), the genomes of *Hadesarchaeota* (9.95%) are more enriched in [Fe-S] binding proteins than are more recently diverging members of this lineage (e.g., type I and II methanogens).

The type I methanogens encode, on average, 123 putative [Fe-S] binding proteins in their respective genomes, while the type II methanogens/methanotrophs encode, on average, ∼15% more (151 versus 123) putative [Fe-S] binding proteins in their respective genomes. As such, 7.05, 6.22, and 7.60%, on average, of the total proteins encoded in the genomes of members of the type I methanogens (*Methanopyrales*, *Methanobacteriales*, and *Methanococcales*, respectively) were predicted to bind [Fe-S] clusters (Table 1 and Fig. 2). Type II methanogens and methanotrophs (*Methanomicrobiales*, *Methanocellales*, and *Methanosarcinales*) encoded smaller percentages (5.34, 5.28, and 5.52%, respectively), on average, of inferred proteins that are predicted to bind [Fe-S] clusters. Although the relative abundance of [Fe-S] proteins within the genomes of type II methanogens is less than that within the type I methanogen genomes, the overall number of [Fe-S] proteins is not similarly decreased. The apparent enrichment of putative [Fe-S] binding proteins in type I versus type II genomes is thus due to the smaller genomes associated with the type I methanogens (average of 1,796 protein-encoding genes) relative to the genomes of type II methanogens (average of 2,845 protein-encoding genes) (Data Set S3).

Archaeal methanogens/methanotrophs/alkanotrophs that branch between type I and type II lineages (*Methanomassiliicoccales* and *Methanonatronarchaeales*) encoded [Fe-S] binding proteins as 5.95 and 5.92% of their inferred proteomes, on average, similar to type I methanogens. Importantly, the average genome sizes of these lineages (1,636 and 1,520, respectively) (Data Set S3) are also more similar to those of type I methanogens. Importantly, when comparing finished versus draft genomes within a taxonomic group, the finished genomes typically contained a higher concentration of putative [Fe-S] binding proteins than the drafts. Only two groups, the *Methanococcales* and *Methanomassiliicoccales*, had higher distributions of these proteins within their draft genomes than within finished genomes. Therefore, although many of the genomes that were analyzed herein are sequence drafts, their [Fe-S] abundance is likely not artificially inflated due to being incomplete. Together, these data suggest a surprisingly similar dependence on [Fe-S] cofactors in type I and type II cells, with unclassified lineages often displaying an increased usage of [Fe-S] clusters. The use of additional iron cofactors, particularly heme, by some methanogens/methanotrophs/alkanotrophs would therefore represent an expansion of overall catalytic capabilities in these cells, rather than simply a shift in cofactor type.

### Distribution of iron-tetrapyrrole biosynthesis pathways.

**FIG 3:**
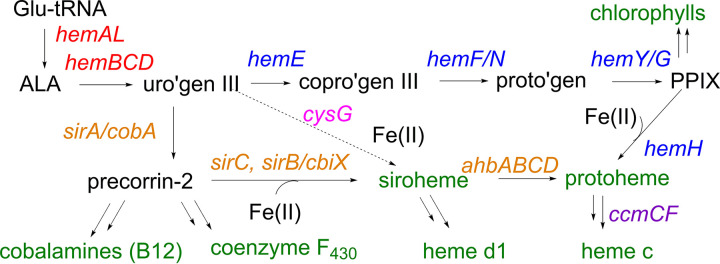
Metabolic pathways for the biosynthesis of structurally related tetrapyrroles in *Archaea* and some *Bacteria*. Cofactors that serve as pathway endpoints are shown in green. *Archaea* use the glutamyl-tRNA pathway for the synthesis of aminolevulinic acid (ALA), which is converted in 3 successive steps to the common intermediate uroporphyrinogen III (uro’gen III; red gene names). Animals, plants, fungi, and some species of *Bacteria* use the canonical heme biosynthetic pathway (blue gene names) to convert uro’gen III first to coproporphyrinogen III (copro’gen III) and then to protoporphyrinogen (proto’gen), which is oxidized by anaerobic (*hemG*) or aerobic (*hemY*) processes to protoporphyrin IX (PPIX). Genes encoding a more ancient, fully anaerobic pathway to synthesize protoheme are highlighted in orange. This pathway proceeds through precorrin-2, a common intermediate leading alternately to the cobalamins, coenzyme F_430_, or siroheme. Siroheme, an Fe-tetrapyrrole-Fe_4_S_4_ cluster conjugate, is converted in four steps to protoheme (*ahbABCD*) and, in *Bacteria*, subsequently cross-linked via its vinyl side chains to *c*-type heme proteins, facilitated by delivery from cytochrome *c* maturation genes (*ccmCF*; purple). The gene-naming convention used here is from Bryant et al. (71).

We next examined the genomic data set for the presence of biosynthetic genes for hemes and precursors of heme belonging to the parent class of compounds, the tetrapyrroles. Tetrapyrroles are a structurally diverse class of metal-binding, macrocyclic ligands that include cofactor F_430_ or F_430_ variants common to archaeal methanogens and methanotrophs, respectively (64–66), as well as cytochromes that are thought to be used primarily by select type II methanogens for electron transfer reactions (30). These cofactors are also likely used by unclassified methanogen/methanotroph/alkanotrophs, considering that those genomes encode MCR and this enzyme is F_430_ dependent (66, 67). Abiotic synthesis of porphyrins and porphyrin-like molecules, the subtype of tetrapyrroles to which the heme macrocycle belongs, has been demonstrated under simulated prebiotic conditions, leading to the suggestion that such molecules have likely existed for at least 4 billion years and, along with [Fe-S] clusters, are likely to be among the most ancient electron transfer cofactors (68–70). Tetrapyrrole biosynthesis has long been an active area of research in *Bacteria* and has more recently become so in *Archaea* (Fig. 3). Enzymes HemBCD, which catalyze the conversion of aminolevulinic acid (ALA) to the precursor uroporphyrinogen, constitute the trunk of the tetrapyrrole biosynthesis pathway from which numerous branches radiate, leading to a variety of (metallo)cofactors at their termini. These include the cobalt-containing cobalamins (including vitamin B_12_), the nickel-containing F_430_ cofactor and variants, the iron-containing siroheme cofactor, magnesium-containing (bacterio)chlorophylls, and iron-containing protoheme cofactors (i.e., iron-protoporphyrin IX, heme *b*, or simply heme) (71).

Until recently, the only known pathway for heme *b* biosynthesis proceeded through protoporphyrin IX (Fig. 3) (72). With increases in genome sequencing, however, it was recognized that several of the genes in this pathway were absent in many *Bacteria* and most *Archaea* (73). An alternative, more ancient pathway to heme *b* with siroheme serving as an intermediate was recently documented in sulfate-reducing bacteria (*Desulfovibrio* sp.) and some *Archaea*, including M. barkeri (74, 75). In these species, precorrin-2 serves as a common precursor to heme *b*, cobalamins, and the Ni-dependent F_430_ cofactor (Fig. 3). Dehydrogenation and metalation of precorrin-2, catalyzed by a precorrin-2 dehydrogenase (SirC) and a chelatase (known alternatively as SirB or CbiX), respectively, or in some cases by a bifunctional enzyme (Met8P), leads to siroheme (76–78). The addition of four fully anaerobic steps, catalyzed by the alternative heme biosynthesis proteins (AhbABCD), yields heme *b* (75).

Given the central role of tetrapyrroles in the metabolism of archaeal methanogens, methanotrophs, and alkanotrophs, we hypothesized that the genomes surveyed would universally possess the common trunk of the tetrapyrrole biosynthetic apparatus, extending from ALA to uroporphyrinogen III and then to precorrin-2 (HemABCD). Precorrin-2 is a necessary precursor for F_430_-dependent methanogenesis. All of the type I methanogens (125/125) encoded homologs of HemBCD (Table 2). Among the class II methanogens/methanotrophs, 99% (167/168) of the genomes encoded HemB, HemC, or HemD. One complete type II genome (Methanofollis liminatans GKZPZ) encoded homologs of only HemBC, and an incomplete MAG affiliated with the *Methanosarcinales* (*Methanomicrobia* archaeon JdFR-19) did not encode HemBCD. Eight of the 10 *Verstraetearchaeota* and 9/11 *Methanomassiliicoccales* genomes encoded HemBCD, with those that did not encode homologs being unfinished genomes. Among the genomes of members of *Bathyarchaeota*, which to date are all incomplete and assembled from metagenomic data, none encoded HemBCD. With the exception of *Archaeoglobales* (1/1) and one *Crenarchaeota* genome (1/4), the early-diverging unclassified genomes affiliated with *Crenarchaeota* and *Hadesarchaeota* did not encode homologs of HemBCD; however, this may be due to the incomplete nature of their genomes, considering the propensity for crenarcheotes to utilize cytochromes (one potential destination for heme) (79). Genomes that lack homologs of HemBCD are unlikely to be capable of synthesizing the intermediate uroporphyrinogen III and, thus, F_430_, cytochromes, or even cobalamin unless alternative, yet-to-be-defined pathways are involved. Given the central involvement of F_430_ (or a structural derivative) as a cofactor for MCR (66, 67) that catalyzes the last step of methanogenesis or the first step of methanotrophy/alkanotrophy (80), one would expect that either these genomes possess an unknown mechanism for performing an equivalent function to HemBCD or these organisms acquire the uroporphyrinogen III intermediate or downstream products from exogenous sources to meet their metabolic demands. Alternatively, the organisms with genomes that lack homologs of HemBCD but encode MCR may not be using MCR for methanogenesis, methanotrophy, or alkanotrophy.

**TABLE 2:** Distribution of protein homologs mediating precorrin-2, siroheme, protoheme (heme *b*), and heme *c* biosynthesis among 326 archaeal methanogen, methanotroph, and alkanotroph genomes

Taxonomic group	Total no. of genomes^*a*^	No. of complete genomes/no. of draft genomes	No. of genomes with indicated protein homologs mediating:					
			Precorrin-2 biosynthesis (HemBCD)^*b*^	Siroheme biosynthesis		Siroheme destination		Protoheme biosynthesis (AhbABCD)
				SirA/CobA	SirC	Sir/Nir	Fsr	
Type I								
* Methanobacteriales*	102	21/81	102	100	99	15	22	0
* Methanococcales*	20	15/5	20	19	20	0	2	0
* Methanopyrales*	3	3/0	3^*c*^	3	3	0	2	0

Type II								0
* Methanocellales*	4	3/1	4	4	4	2	0	0
* Methanomicrobiales*	32	11/21	32	25	28	11	0	0
* Methanosarcinales*	132	37/95	131	131	122	121	8	114

Unclassified								
* Archaeoglobales*	1	0/1	1	1	1	1	0	0
* Bathyarchaeota*	3	0/3	0	1	0	0	0	0
* Crenarchaeota*	4	0/4	1	1	1	0	0	0
* Hadesarchaeota*	2	0/2	0	0	0	0	0	0
* Methanomassiliicoccales*	12	4/8	9	9	2	0	0	0
* Methanonatronarchaeales*	1	0/1	1	1	1	0	0	1
* Verstraetearchaeota*	10	0/10	8	9	6	0	0	0

a The number of genomes within a defined taxonomic group that encoded one or more homologs is indicated. Searched sequences (Data Set S2), the distribution of homologs and their accession numbers among the 326 individual genomes (Data Set S3), and information about the searched genomes (Data Set S1) are provided in the supplemental material.

b Genomes were scored as positive if homologs of HemB, HemC, or HemD were encoded in the genome, to account for incomplete pathways in draft genomes.

c *Methanopyrales* genomes encoded a homolog of HemD outside our BLAST threshold parameters.

Subsequent conversion of the HemBCD product uroporphyrinogen III to precorrin-2 and then siroheme occurs in three additional steps that involve the SirABC biosynthesis proteins (Fig. 3). Homologs of SirB were not compiled as part of this study, since they cannot be accurately demarcated from paralogs using primary sequences alone. Nonetheless, homologs of SirA and SirC were identified in 119/125 (95% of total) and 154/169 (91%) of the type I and type II genomes examined (Table 2), with instances of their absence generally associated with incomplete genomes (Table S3). Across all of the 326 genomes examined, there was a strong correspondence between the distribution of SirA and SirC homologs in genomes (Data Set S3), consistent with their concerted role in siroheme biosynthesis. Homologs of SirA and SirC could not be identified in a number of unclassified genomes, suggesting either an inability to use siroheme or incompleteness in the genomic data (Table 2). We therefore looked concurrently for evidence of siroheme usage, via expected siroheme binding motifs and proteins.

### Evidence for (siro)heme usage.

Contrary to the catalytically versatile protoheme cofactor (discussed below), siroheme has been found in only two enzymes to date, nitrite and sulfite reductases, where it catalyzes the 6-electron reduction of the anion (7, 81). Sulfite is a product of dissimilatory or assimilatory sulfate reduction and a potent inhibitor of methanogenesis. In recent surveys of archaeal genomes (5, 82), siroheme-associated *dsr*- or *fsr-*like genes (encoding F_420_-independent or -dependent sulfite reductases, respectively) were found only in the methanogens. Consequently, we examined our genomic database for the presence of homologs of these proteins as a way of further ascertaining siroheme usage. Because *dsr*-encoded sulfite and nitrite reductases are homologous, we expected the searches for Dsr homologs presented here to identify either or both enzymes. Among the type I methanogens surveyed, nearly 69% (86/125) of the genomes, many of them complete, lacked a homolog of either Fsr or Dsr in spite of possessing siroheme biosynthetic enzymes SirA and SirC (Table 2). This may suggest other, unknown protein destinations for siroheme in these cells. Similarly, all of the unclassified genomes lacked Fsr/Dsr homologs, with the exception of the representative species from *Archaeoglobales*. In contrast, among type II genomes, homologs of Dsr were prevalent. Nearly all (121/132) *Methanosarcinales* genomes encoded Dsr homologs. The few identified homologs of Fsr were found only among *Methanosarcinales* genomes, and with the exception of a single incomplete genome, those that encoded Fsr also encoded homologs of SirAC. In every case, the Fsr and Dsr homologs identified all possessed the expected, distinctive siroheme binding motif (CX4-5CXnCX3C) (Data Set S4), confirming that the proteins likely do bind siroheme.

Modification of siroheme via the activities of 4 proteins (AhbABCD) leads to heme *b*. The only genomes where homologs of AhbABCD were identified were among type II methanogens (Table 2), in particular those affiliated with *Methanosarcinales* and one member of the *Methanomassiliicoccales*. None of the unclassified genomes encoded homologs of AhbABCD. This distribution is consistent with previous reports of a limited distribution of cytochromes among methanogens, most notably among members of the *Methanosarcinales* (83–86). Heme-synthesizing capabilities would seem to offer substantial evolutionary benefits, since heme is a catalytically versatile cofactor associated with a number of methanogen proteins, including a cytochrome-containing and membrane-bound [NiFe] hydrogenase (VhoC) and a cytochrome-containing heterodisulfide reductase (HdrE) that catalyze the first and last steps of methanogenesis from CO_2_ and H_2_, respectively. As summarized by Thauer et al. (30), in methanogens without cytochromes, these first/last steps are energetically linked through an electron-bifurcating [NiFe] hydrogenase-heterodisulfide reductase complex, whereas in cytochrome-containing methanogens (e.g., *Methanosarcinales*), the same steps are coupled chemiosmotically, allowing for much higher energy yields at the expense of being less competitive for growth at lower H_2_ partial pressures. The ability among methanogens to synthesize cytochromes is thus expected to be selectable in environments where H_2_ is at high concentrations.

The benefits of heme have led to its uptake by many bacterial anaerobes that are incapable of synthesizing it. Many lactic acid bacteria, for example, benefit from or in some cases depend on environmentally derived heme (87). We hypothesized that both the heme-synthesizing *Methanosarcinales* and some of the apparent nonsynthesizers identified here could benefit from heme usage and looked for evidence for heme auxotrophy via several parallel strategies. First, we searched for homologs of heme *b*-binding proteins with well-documented biological roles in methanogens: the VhoC and HdrE enzymes described above. The distribution of these proteins hewed closely to established expectations, as they were found exclusively among heme *b*-synthesizing type II species (76% and 93% of the *Methanosarcinales*, respectively) (Table 3), where their adoption may reflect the selective advantages described above.

**TABLE 3:** Distribution of protoheme (heme *b*) motifs or proteins that putatively bind heme *b* among 326 archaeal methanogen, methanotroph, and alkanotroph genomes

Taxonomic group	Total no. of genomes^*a*^	No. of complete genomes/no. of draft genomes	No. of genomes with heme motif or protein destination				
			Heme *b* motif^*b*^	VhoC	HdrE	KatG	Cyt *bd* oxidase
Type I							
* Methanobacteriales*	102	21/81	13	0	0	13	0
* Methanococcales*	20	15/5	0	0	0	0	0
* Methanopyrales*	3	3/0	0	0	0	0	0

Type II							
* Methanocellales*	4	3/1	2	0	0	1	0
* Methanomicrobiales*	32	11/21	8	0	0	7	0
* Methanosarcinales*	132	37/95	117	87	106	32	24

Unclassified							
* Archaeoglobales*	1	0/1	1	0	0	1	0
* Bathyarchaeota*	3	0/3	0	0	0	0	0
* Crenarchaeota*	4	0/4	0	0	0	0	0
* Hadesarchaeota*	2	0/2	0	0	0	0	0
* Methanomassiliicoccales*	12	4/8	1	1	1	0	0
* Methanonatronarchaeales*	1	0/1	0	0	0	0	0
* Verstraetearchaeota*	10	0/10	2	0	0	2	0

a The number of genomes within a defined taxonomic group that encoded one or more homologs is indicated. Searched sequences (Data Set S2), the distribution of homologs and their accession numbers among the 326 individual genomes (Data Set S3), and information about the searched genomes (Data Set S1) are provided in the supplemental material.

b Number of genomes that encoded one or more proteins with a putative heme *b* binding motif, GX(H/R)XCXGX.

We next searched for examples of heme *b*-dependent proteins shown in a widescale study of diverse bacterial genomes to be broadly distributed in aerotolerant anaerobes (88), since these might also be prevalent in aerotolerant archaeal methanogens, methanotrophs, and alkanotrophs. KatG is a heme *b*-dependent catalase-peroxidase that is known to be critical for H_2_O_2_ detoxification in aerobes (89) and has been identified in multiple anaerobes (including Methanosarcina acetivorans) (90). Cytochrome *bd* oxidase (Cyt *bd*) is a terminal respiratory oxidase with high affinity for extremely low quantities of O_2_ in the obligate anaerobic bacteria Bacteroides fragilis and Desulfovibrio gigas (91, 92). Homologs of these proteins were surprisingly prevalent among the genomic database examined here (Table 3). For example, 13/102 *Methanobacteriales* (type I), 7/32 *Methanomicrobiales* (type II), and 32/132 *Methanosarcinales* (type II) possessed a KatG homolog. In the case of M. barkeri (type II) (93) and Methanobrevibacter arboriphilus AZ and DH1 (type II) (94), functional catalase has been identified and characterized. Furthermore, 24 of the 132 *Methanosarcinales* genomes encode homologs of Cyt *bd* (Table 3). The presence of homologs of these proteins in these genomes, if they are indeed functional, suggests that these species have at least some exposure to O_2_, perhaps as a detoxification strategy. They also suggest the possible use of environmentally derived heme in the case of the type I species, the type II *Methanomicrobiales*, and potentially, some of the *Methanosarcinales*, since these do not appear to possess complete AhbABCD pathways for its biosynthesis.

Third, we used a custom hidden Markov model (HMM) to identify proteins that encode a heme *b* binding motif (GX[H/R]XCXGX) (Data Set S4) in the genomic database. In general, the output of the custom script and individual BLASTp searches for VhoC, HdrE, KatG, and CytBD were congruent (Table 3; Data Sets S3 and S4), pointing to the efficacy of the script to identify proteins with this motif. All of the motifs identified were associated with sequences for VhoC, HdrE, KatG, or CytBD proteins (data not shown). The inability to identify proteins outside this limited set could reflect the diversity of sequences and folds that accommodate heme *b* and the limitations of using a custom script developed based on the characterized heme *b* binding motif.

Finally, we tested the hypothesis that, like anaerobic *Bacteria*, the archaeal methanogens, methanotrophs, and alkanotrophs investigated here might benefit from the electron-shuttling heme *c* cofactor, in which the heme is covalently cross-linked via its vinyl side chains to a pair of cysteine residues in the target protein, frequently but not exclusively arranged in a canonical CXXCH motif. Indeed, the majority of the *Methanosarcinales* genomes (99/114) that encoded core essential genes for heme *b* biosynthesis (Table 3) also encoded homologs of one or more cytochrome *c* maturation (Ccm) proteins (Table 4) (95, 96). In *Bacteria*, these proteins are collectively responsible for the transport (CcmAB), delivery (CcmCEF), or oxidative cross-linking (CcmGH) of heme *b* to it target proteins (97, 98). In addition to the *Methanosarcinales*, several species lacking the AhbABCD protein also encode homologs of CcmCF, including the single *Methanonatronarchaeales* genome.

**TABLE 4:** Distribution of proteins among genomes with heme *c* motifs or proteins that putatively bind heme *c* among 326 archaeal methanogen, methanotroph, and alkanotroph genomes

Taxonomic group	Total no. of genomes^*a*^	No. of complete genomes/no. of draft genomes	No. of genomes with heme *c* motif or destination										
			Heme *c* motif^*b*^	CcmCF	NarI	CcdA	CcP	MauG	Cbb3	TorA	CytC	NrfA	MsmS
Type I													
* Methanobacteriales*	102	21/81	0	0	0	0	0	0	0	0	0	0	0
* Methanococcales*	20	15/5	0	0	0	0	0	0	0	0	0	0	0
* Methanopyrales*	3	3/0	0	0	0	0	0	0	0	0	0	0	0

Type II													
* Methanocellales*	4	3/1	0	0	0	0	0	0	0	0	0	0	0
* Methanomicrobiales*	32	11/21	5	0	0	1	0	0	0	0	0	0	4
* Methanosarcinales*	132	37/95	94	99	1	0	5	1	1	64	81	3	19

Unclassified													
* Archaeoglobales*	1	0/1	1	1	0	0	0	0	0	0	0	0	1
* Bathyarchaeota*	3	0/3	0	0	0	0	0	0	0	0	0	0	0
* Crenarchaeota*	4	0/4	0	0	0	0	0	0	0	0	0	0	0
* Hadesarchaeota*	2	0/2	0	0	0	0	0	0	0	0	0	0	0
* Methanomassiliicoccales*	12	4/8	1	0	0	0	0	0	0	1	1	0	0
* Methanonatronarchaeales*	1	0/1	0	1	0	0	0	0	0	0	0	0	0
* Verstraetearchaeota*	10	0/10	0	0	0	0	0	0	0	0	0	0	0

a The number of genomes within a defined taxonomic group that encoded one or more homologs is indicated. Searched sequences (Data Set S2), the distribution of homologs and their accession numbers among the 326 individual genomes (Data Set S3), and information about the searched genomes (Data Set S1) are provided in the supplemental material.

b Number of genomes that encoded one or more proteins with putative heme *c* binding motif CXXCH, CPV, CX_15_CH, or CXXCK.

In parallel, we searched the genome database for proteins encoding the consensus heme *c* binding motif, CXXCH, as well as heme *c* binding motifs CPV, CX_15_CH, and CXXCK, using a custom Python script (Materials and Methods and Data Set S4). Proteins with heme *c* binding motifs were identified among the *Methanomicrobiales* (5/32 genomes), *Methanosarcinales* (94/132 genomes), and *Methanomassiliicoccales* (1/12 genomes) (Table 4). The sole *Archaeoglobales* genome encoded Ccm and proteins with heme *c* binding motifs. Proteins identified with a heme *c* binding motif included a variety of known heme *c* binding proteins, such as subunits of the respiratory tetraheme-trimethylamine oxidase (TorA) and cytochrome *c*_522_-dependent nitrate reductases (NarI), cytochrome *c* peroxidase (CcP), cytochrome *c* biogenesis protein (CcdA), the diheme tryptophan tryptophylquinone oxygenase (MauG), respiratory cytochrome *d* and *bb*_3_ oxidases (CydAB and Cbb3), cytochrome *c* (CytC), cytochrome *c*_552_-dependent nitrite reductase (NrfA), and the heme-dependent dimethyl sulfide sensor (MsmS). MsmS proteins in M. acetivorans only bind heme via a single cysteine (99), and the homologs identified herein contained either a CXXCH or CXXXAH motif (data not shown). Full sequences of the proteins identified above were subsequently used as bait in subsequent BLASTp searches of the 326 genomes. In general, the outcome of the HMM was similar to the outcome of BLASTp, identifying homologs overwhelmingly among the *Methanosarcinales* but with a few notable exceptions from four *Methanomicrobiales* (type II) genomes that encoded MsmS homologs and a single *Methanomassiliicoccales* genome (unclassified archaeon ISO4-G1) that encoded homologs of TorA and CytC. Collectively, these results suggest that, while heme biosynthesis and the preponderance of known pathways for heme usage are largely confined to members of the *Methanosarcinales*, other type I, type II, and unclassified archaeal methanogens, methanotrophs, and alkanotrophs likely encode heme-dependent processes. Moreover, the diversity of processes where heme *b* or *c* is used, even within the *Methanosarcinales*, clearly extends beyond methanogenesis-associated processes like those catalyzed by VhoC and HdrE, and these remain to be explored physiologically and biochemically.

### Iron and sulfur storage.

Finally, the database of 326 genomes was examined for proteins associated with storage of iron, sulfur, or both, beginning with ferritins. Proteins from the ferritin superfamily are ubiquitous in all kingdoms of life (100). In O_2_-utilizing species, members of the family form 24-homooligomeric capsules that serve as vesicles for storage of Fe as ferric oxide mineral nanoparticles. Even in some anaerobic bacteria, ferritin superfamily proteins have been shown to hold iron oxide deposits, where the ferritin particle is presumed to function in detoxifying Fe(II)/O_2_. Mechanisms for reductive mobilization and release of the Fe have so far not been described (101). Fe(II) is oxidized by O_2_ as it crosses the threshold of the ferritin nanocompartment, at a catalytic site that itself contains heme (in the case of bacterioferritin, Bfr) or a nonheme di-iron cofactor (in the ferritin A protein, FtnA) (102). Ferritin family proteins have additionally been ascribed roles in transcriptional regulation, which will not be further addressed here (103).

Homologs of either Bfr or FtnA proteins were identified in 112/125 type I methanogens, where the overwhelming majority (104/112) were of the nonheme, FtrA type (Table 5). The absence of homologs of Bfr and FtnA among some type I methanogens is unlikely to be an artifact of genome incompleteness, since at least 8 genomes that lacked homologs of either protein were complete. Eight genomes, largely affiliated with the genus *Methanobrevibacter* (7), encoded Bfr. Seven of these 8 genomes also encoded a homolog of FtrA. Approximately half of the *Methanomicrobiales* (16/32 genomes) possessed homologs for FtrA, while 51/132 and 89/132 *Methanosarcinales* genomes encoded FtrA and Bfr, respectively. Thirteen *Methanosarcinales* genomes encoded homologs of both proteins. The apparent prevalence of heme-containing Bfr homologs among *Methanosarcinales* genomes, but not other type I and II genomes, is consistent with the presence of homologs of heme biosynthesis pathways in these organisms (Table 2).

**TABLE 5:** Distribution of homologs of bacterioferritin, ferritin, and the iron sulfur storage protein IssA among 326 archaeal methanogen, methanotroph, and alkanotroph genomes

Taxonomic order	Total no. of genomes^*a*^	No. of complete genomes/no. of draft genomes	No. of genomes with indicated protein homologs^*b*^		
			FtnA	Bfr	IssA^*c*^
Type I					
* Methanobacteriales*	102	21/81	91	8	16
* Methanococcales*	20	15/5	13	0	14
* Methanopyrales*	3	3/0	0	0	3

Type II					
* Methanocellales*	4	3/1	1	4	0
* Methanomicrobiales*	32	11/21	16	0	24
* Methanosarcinales*	132	37/95	51	89	110

Unclassified					
* Archaeoglobales*	1	0/1	1	0	0
* Bathyarchaeota*	3	0/3	0	0	0
* Crenarchaeota*	4	0/4	0	4	0
* Hadesarchaeota*	2	0/2	0	0	1
* Methanomassiliicoccales*	12	4/8	0	3	6
* Methanonatronarchaeales*	1	0/1	0	0	0
* Verstraetearchaeota*	10	0/10	1	0	0

a The number of genomes within a defined taxonomic group that encoded one or more homologs is indicated. Searched sequences (Data Set S2), the distribution of homologs and their accession numbers among the 326 individual genomes (Data Set S3), and information about the searched genomes (Data Set S1) are provided in the supplemental material.

b FtnA, ferritin (nonheme dependent); Bfr, bacterioferritin (protoheme dependent); IssA, iron-sulfur storage protein.

c The IssA homologs retrieved were further analyzed according to methods described by Vaccaro et al. (104) in order to assess their likely membership in IssA versus NifB and NafY families, as shown in Fig. S2.

Curiously, all of the *Methanocellales* and *Crenarchaeota* also encoded homologs of Bfr (Table 5), despite an apparent inability to synthesize heme (Table 2). It is possible that these species incorporate and use exogenous heme obtained from their environments. It is also possible that ferritin superfamily proteins have as-yet-unidentified roles in *Archaea*, which may not depend on protoheme binding or involve exposure of these species to O_2_. The biological roles of ferritins in extant archaeal species, in short, remain unclear. Additional work is clearly warranted to determine how Fe is stored/detoxified in FtrA- and Bfr-like homologs among archaeal methanogens, methanotrophs, and alkanotrophs, all of which are anaerobes, and whether O_2_ is involved in this process.

Storage of Fe in the ferrous state or a mixed ferrous/ferric state would seem to be better strategies for an anaerobe, particularly one with adequate protections against occasional, unwanted exposure to O_2_, such as with catalases (e.g., KatG) or peroxidases. Storage of Fe(II) or Fe(II)/Fe(III) could occur in the form of the [Fe-S] clusters themselves, particularly given their high apparent usage and abundance in methanogens, methanotrophs, and alkanotrophs. Alternatively, Fe(II)/Fe(III) might be reversibly stored in mineralized form in association with a protein scaffold that functionally mirrors ferritin. In 2017, evidence for such a scenario was provided by Vaccaro et al. with the discovery of the iron-sulfur storage protein (IssA) in Pyrococcus furiosus (104). This protein was able to reconstitute the [Fe-S] clusters of ferredoxin *in vitro* and scaffolded the formation of a thioferrate nanomineral species *in vivo*, hence satisfying the requirements of a true storage mechanism (104). The authors identified IssA homologs in numerous bacterial and archaeal genomes, including methanogens, where they were sorted into probable functional clades based on phylogenetic relationships. These clades included IssA and the NifB, NifX, and NafY protein families that function in the maturation of the iron-molybdenum-sulfur cluster used by nitrogenase, among other roles (105, 106). In many organisms, NifB represents a fusion of an N-terminal radical *S*-adenosylmethionine (SAM) domain protein (NifB) and a C-terminal NifX-like domain protein (107); in some cases, the radical SAM domain protein and the NifX protein are encoded by separate genes termed *nifB* and *nifX*, respectively. The C-terminal half of NafY exhibits high sequence similarity to NifX (106), and the C-terminal region of IssA is homologous to NifX (104). As such, all of these proteins have a shared NifX domain (InterPro globular domain IPR003731 [108]), and like the P. furiosus homolog (PF2025) (104), this may serve as a scaffold for the assembly and storage of simple or complex [Fe-S] clusters or thioferrate-like compounds.

Using the P. furiosus IssA homolog (PF2025) as a BLASTp query, we compiled IPR003731 globular domain proteins and grouped them based on phylogenetic reconstructions as either NifX, NafY, NifB, or IssA homologs based on characterized representative proteins, as described previously (Fig. S2) (104). Homologs of putative IssA proteins were thereby identified in the majority of type II species (134/168) but a much lower proportion of type I species (33/125) (Table 5). This was unexpected, since type I methanogens are more similar to P. furiosus, the organism where IssA was first discovered (104). In contrast, 104/125 type I species contain an FtnA homolog, though it is unclear how these organisms would use an apparently O_2_-dependent mechanism for iron storage. The biological roles of these proteins and the means by which Fe is stored therefore require further genetic and physiological study.

### Integration of phylogenomic and gene distribution data.

**FIG 4:**
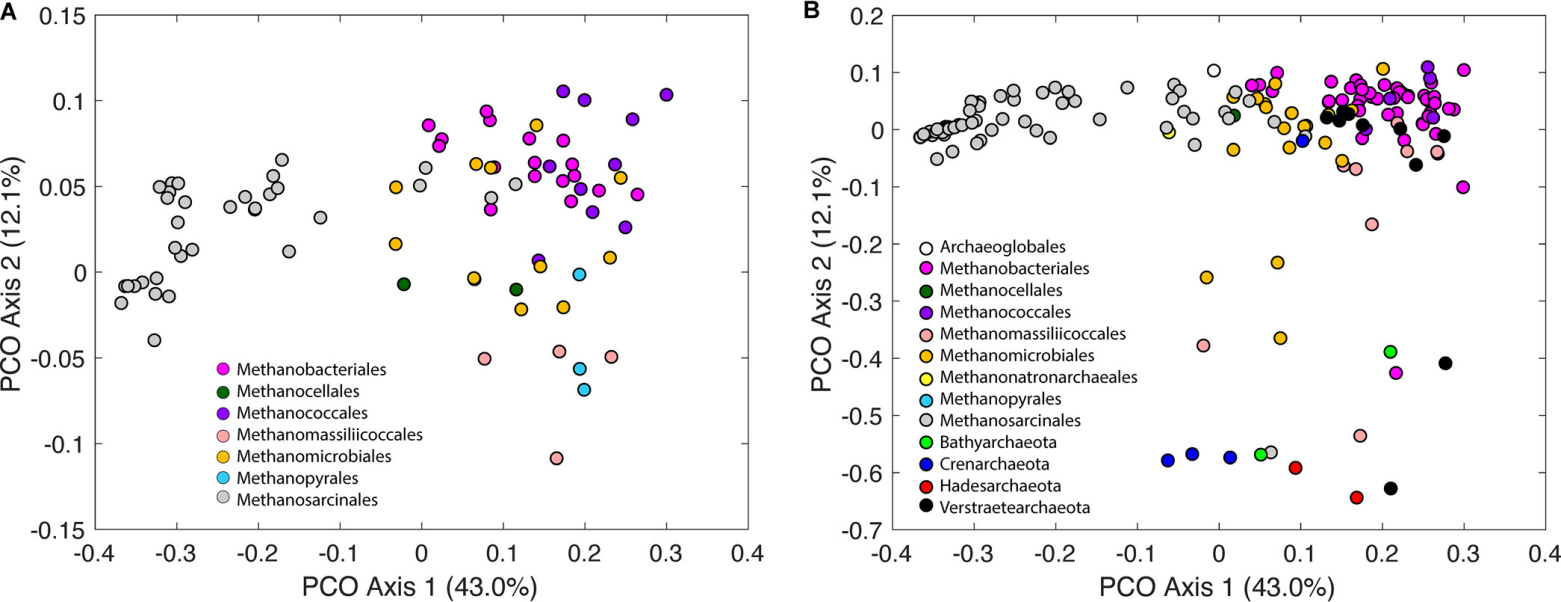
Principal coordinate ordination (PCO) of a matrix describing the Jaccard dissimilarity in the distribution of protein homologs involved in the acquisition, trafficking, destination, and storage of iron and sulfur among 326 archaeal methanogens, methanotrophs, and alkanotrophs. The same PCO ordination is presented for complete (A) and incomplete (B) genomes. Construction of the matrix did not include the distribution of proteins with heme *b*, heme *c*, or [Fe-S] cluster binding motifs unless they were particular targets of BLASTp analyses of the genomes (Data Set S3). The taxonomic group designations are represented by a color overlay on the symbols.

One of the goals of this study was to determine how Fe/S uptake and deployment varied among classified (types I and II) and unclassified archaeal methanogens, methanotrophs, and alkanotrophs. Thus, the distribution of homologs of proteins involved in these functionalities (Tables 1 to 4; Data Set S3) was used to create a dissimilarity matrix for use in comparing the composite of these proteins among the 326 complete and incomplete genomes comprising the database. A principal coordinate ordination (PCO) of this matrix for complete genomes (Fig. 4A) revealed trends that generally corresponded to the taxonomic and evolutionary groupings of these genomes. The majority of the variance in the distribution of protein homologs among genomes was explained by PCO axis 1 (43.0% of variance explained) and, to a lesser extent, PCO axis 2 (12.1% of variance explained). PCO axis 1 generally separated type I genomes and *Methanosarcinales* genomes, with the majority of type I genomes (*Methanobacteriales*, *Methanococcales*, and *Methanopyrales*) plotting in the upper right quadrant of the PCO. While most type II *Methanosarcinales* genomes formed a cluster in the left quadrant of the PCO, the other type II genomes (*Methanocellales* and *Methanomicrobiales*) generally plotted intermediate to the type I genomes and the type II *Methanosarcinales* genomes along both PCO axes 1 and 2. With few exceptions (noted below), the clustering of type I and II genomes based on taxonomy was generally insensitive to those genomes being complete or incomplete (Fig. 4A and B). The patterns of clustering of type I and type II genomes along PCO axis 1 are therefore largely attributed to differences in the distribution of protein homologs involved in synthesizing heme *b* (AhbABCD) (Table 2) and heme *c* (CcmCF) (Table 4), proteins that utilize heme *b* (VhoC, HdrE, and KatG) (Table 3) and heme *c* (TorA, CytC, and MsmS) (Table 4), and to a lesser extent, siroheme destination (Dsr and Fsr) (Table 2).

Several type I and type II genomes clustered distinctly from others in these taxonomic groupings along PCO axis 2 (12.1% variance explained). In particular, one *Methanobacteriales* (*Methanobrevibacter* sp. RUG833), one *Methanosarcinales* (*Methanomicrobia* archaeon JdFR-19), and three *Methanomicrobiales* genomes (*Methanomicrobiales* archaeon Methan_05, 06, and 07) cluster distinctly from other members of their higher-order taxonomic groups (Fig. 4B). All of these genomes are incomplete, suggesting that clustering along PCO axis 2 may be an artifact of genome completeness. In potential support of this is the general observation that nearly all of the *Bathyarchaeota* and *Hadesarchaeota* and many of the *Crenarchaeota* and *Verstraetearchaeota*, which are all incomplete, plot distinctly from the other type I and II genomes along PCO axis 2 (Fig. 4B). And yet, several unclassified genomes that are also incomplete cluster with type I and type II genomes along PCO axis 2, namely, a representative of the *Crenarchaeota* (unclassified *Crenarchaeota* GMQP bin_37), a representative of the *Archaeoglobales* (unclassified *Archaeoglobales* GMQP bin_32), and six of the eight *Verstraetearchaeota* genomes. In general, the unclassified *Archaeoglobales* GMQP bin_32 clusters along PCO axis 1 more closely to type II genomes, while the unclassified *Crenarchaeota* GMQP bin_37 and six of the eight *Verstraetearchaeota* genomes cluster more closely with the type I genomes.

Importantly, 10 of the 12 *Methanomassiliicoccales* genomes, including both those that are complete and incomplete, clustered with type I genomes along PCO axes 1 and 2. The two that did not cluster with the type I and type II genomes along PCO axis 2 were both incomplete genomes. These same 10 genomes all plotted with type I methanogens along PCO axes 1 and 2. The sole unclassified *Methanonatronarchaeales* genome, which was also incomplete, plotted with the type II genomes along both PCO axes 1 and 2. Together, these observations suggest that while true differences likely exist in the clustering of unclassified genomes along PCO axis 2 based on the distribution of protein homologs involved in Fe/S uptake and deployment, it is possible that some amount of the clustering along PCO axis 2 is due to genome incompleteness.

We next sought to compare patterns of clustering in the PCO based on Fe and S metabolism (Fig. 4A and B) with the phylogenetic relationships of the 326 archaeal methanogen, methanotroph, and alkanotroph genomes (Fig. 3; Fig. S1). The sole *Methanonatronarchaeales* genome and the unclassified *Archaeoglobales* genome, which cluster phylogenomically with type II methanogen/methanotroph genomes, also harbor a similar distribution of protein homologs involved in the acquisition, trafficking, destination, and storage of Fe and S. This suggests that they could be grouped within the current scheme as type II, at least on the basis of Fe and S metabolism. Members of the unclassified *Methanomassiliicoccales*, which form a lineage basal to other type II methanogen and methanotroph genomes and sister to more recently evolved type I genomes (*Methanococcales*/*Methanobacteriales*), share more similarity with type I genomes at the level of the distribution of protein homologs involved in Fe/S uptake and deployment. Thus, members of the *Methanomassiliicoccales* could be grouped within the current classification scheme as type I.

Reconciling the phylogenetic relationships and pattern of clustering in the PCO among more deeply diverging unclassified methanogen and alkanotroph genomes is a bit less straightforward. All *Bathyarchaeota*, *Crenarchaeota*, and *Verstraetearchaeota* genomes form a lineage separate from type I and II methanogens/methanotrophs (Fig. 3; Fig. S1). *Hadesarchaeota* genomes branch at the base of the primary lineage that comprises type I and II methanogens/methanotrophs. The *Hadesarchaeota* and *Bathyarchaeota*, 2/8 *Verstraetearchaeota*, and 3/4 *Crenarchaeota* genomes cluster in the PCO ordination, consistent with their clustering deep in the phylogeny (Fig. 3; Fig. S1). The 6 *Verstraetearchaeota* and the single *Crenarchaeota* genome that do not cluster on the PCO ordination with members of their higher-order designations tend to encode components of precorrin-2 biosynthesis (Data Set S3), whereas the others do not. As mentioned above, it is possible that this is due to genome incompleteness. It is also possible that these genes were acquired in these select genomes through horizontal gene transfer or that they were lost. In several cases (e.g., HemA in unclassified *Crenarchaeota* GMQP bin_37), the closest BLASTp hit from unclassified genomes that encode precorrin-2 biosynthesis homologs is to homologs in type I methanogen genomes, suggesting the possibility of horizontal gene transfer. Additional sequencing and more robust genome assemblies will be required to fully elucidate the Fe/S uptake and deployment of these strains. Their clustering on the PCO along axis 1, which is right shifted, is consistent with them being more similar in this respect to type I methanogens than to type II archaeal methanogens and methanotrophs.

### Conclusions.

Methanogens, and by extension archaeal methanotrophs and alkanotrophs that share similar metabolic backgrounds and ecologies, have a high demand for Fe and S to meet their needs for electron-mobilizing cofactors. The comprehensive survey of 326 methanogen, methanotroph, and alkanotroph genomes presented here suggests a general framework for Fe/S uptake and deployment in these species (Fig. 1), in which Fe and S are assimilated as Fe(II) (FeoAB) and cysteine (SufS). These elements are then assembled into [Fe-S] clusters by a truncated SufBC system or, to a far lesser extent, into siroheme (HemBCD and SirABC) and/or heme (AhbABCD) and potentially stored or detoxified on mineral-nucleating protein scaffolds (FtrA, Bfr, and IssA). However, while elements of this generalized picture are true for many, particularly type II species, the picture is also riddled with important exceptions. For example, while possession of the SufBC scaffold appeared to be close to universal, FeoAB was not, nor were the source of sulfide (SufS) or the putative sulfide and Fe(II) chaperones associated with the Suf system (SufD and SufE), suggesting the existence of yet-undescribed mechanisms for the import and intracellular trafficking of these elements. One intriguing source of Fe and S capable of supporting methanogens is iron sulfides, such as FeS and FeS_2_, that are common in anoxic environments (43). Even more surprising, an unexpected diversity of proteins associated with heme usage and/or modification were identified, both within the heme-synthesizing *Methanosarcinales* and in phylogenetically diverse groups that lack the necessary enzymes (AhbABCD) for making heme, while many siroheme-synthesizing species had no apparent siroheme binding proteins. This suggests yet-unknown siroheme and heme biochemistry in these species. The prevalence of ferritin superfamily proteins (FtrA or Bfr) versus the somewhat restricted distribution of the iron sulfide-mineralizing IssA protein, particularly among type I species, begs the question of whether and how these proteins are involved in iron storage or detoxification in the anaerobes that possess them. The observations presented here point toward numerous avenues for follow-up work, among both the better-characterized model type I and II species and the ancient and exceedingly mysterious groups of organisms that fall outside these classifications.

While the majority of the genomes of unclassified archaeal methanogens, methanotrophs, and alkanotrophs are incomplete and thus prevent a definitive assessment of proteins involved in Fe/S acquisition, trafficking, and destination, the abundance of proteins with putative [Fe-S] binding motifs among their genomes was similar to that of the more deeply diverging type I cells. Similarly, the distribution of proteins involved in the biosynthesis and utilization of heme and siroheme was fairly similar. While further work is clearly warranted to determine how these unclassified cells differ from their more well characterized type I and II methanogen/methanotroph counterparts, the observations presented herein suggest them to be more similar to type I cells than type II cells at the level of Fe/S acquisition, trafficking, and use.

## MATERIALS AND METHODS

### Compilation of genomes of putative archaeal methanogens, methanotrophs, and alkanotrophs.

Publicly available genomes from putative methane-/alkane-metabolizing taxonomic groups were compiled from the Joint Genome Institute Integrated Microbial Genomes and Microbiomes (JGI-IMG/MER; https://img.jgi.doe.gov/cgi-bin/mer/main.cgi) for use in phylogenomic reconstruction and informatics analyses (see Data Set S1 in the supplemental material). This included metagenome-assembled genomes (MAGs) from taxonomic groups that have been implicated either through metabolic reconstruction (e.g., the presence of MCR) or physiological experimentation to be involved in methane or alkane metabolism. Phylogenomic reconstruction of the archaeal genomes or MAGs was conducted by surveying them for 103 archaeon-specific single-copy housekeeping phylogenetic-marker genes with Amphora2 (109). Marker genes comprising single-copy, universal archaeal housekeeping genes were included in the analysis, including those encoding 41 ribosomal proteins in addition to RNA polymerase subunits, tRNA synthetase subunits, and other core physiological functional proteins, as previously described (109). Individual protein coding genes were compiled, aligned with Clustal Omega (version 1.2.4) (110), and then concatenated into a supermatrix comprising 56,689 alignment positions. MAGs estimated to be <50% complete based on housekeeping gene complements, as estimated using CheckM version 1.0.7 (111), were excluded from further phylogenomic and bioinformatics analyses. The 326 genomes and MAGs that met this criterion are listed in Data Set S1. The concatenated alignment was then subjected to maximum-likelihood phylogenetic analysis using IQ-TREE (version 1.6.11) (112) with the LG+G amino acid substitution model. Node support was evaluated with 1,000 ultrafast bootstraps (113). Genomes were assigned to phyla and were categorized as type I (*Methanobacteriales*, *Methanococcales*, and *Methanopyrales*) and type II (*Methanomicrobiales* and *Methanosarcinales*), based on previous classification schemes (8, 10, 12). Genomes from putative methanogens, methanotrophs, and alkanotrophs that did not fit within this earlier classification scheme (i.e., *Archaeoglobales*, *Bathyarchaeota*, *Crenarchaeota*, *Hadesarchaeota*, *Methanomassiliicoccales*, *Methanonatronarchaeales*, and *Verstraetearchaeota*) were labeled as unclassified.

To evaluate the accuracy in topological structure between the phylogeny of the select 326 genomes in our database against an *Archaea*-wide database, a genomic phylogeny was also constructed using 2,337 representative genomes from the recently published Genome Taxonomy Database (GTDB) (29). The *Archaea*-wide phylogeny was reconstructed from the GTDB multiple-sequence alignment (5,125 alignment positions) using the phylogenetic methods described above. Many of the protein-coding genes involved in the GTDB multiple sequence alignment are also present in the phylogenetic analysis described above for the 103 universal archaeal housekeeping genes. The GTDB phylogeny is presented in Fig. S1.

### Genomic distribution of protein homologs putatively involved in Fe acquisition and (siro)heme or [Fe-S] cluster biosynthesis.

Unless otherwise stated, protein homologs putatively involved in Fe acquisition and metabolism were identified by BLASTp analyses of the compiled genomic database using query protein sequences from Methanosarcina acetivorans C2A (NCBI Taxonomy identification number [ID] 2214) (see descriptions of query sequences and BLASTp search sequence identifiers in Data Set S2). These included homologs of FeoAB (AAM06845 and AAM06843, respectively) involved in Fe(II) uptake (114) and homologs of the essential enzymes specific to the archaeal/alternative heme biosynthetic pathway AhbABCD (AAM04018, AAM04019, AAM06408, and AAM04017, respectively) (72, 115). Homologs of the membrane protein CcmC, responsible for delivery of protoheme to the cytoplasmic chaperone CcmE, and CcmF, required for the transfer of heme from CcmE to apocytochrome *c* for late cytochrome *c* maturation by acting as a heme lyase (116, 117), were also examined using representative CcmCF proteins (AAM04843 and AAM06674, respectively). Candidate CcmCF proteins were screened for the conserved tryptophan-rich motif in the periplasmic domain that characterizes these proteins (96, 117). IMG gene identifiers for all homologs identified in this study are provided in Data Set S3.

Homologs of proteins common to the biosynthetic pathways for multiple tetrapyrroles or tetrapyrrole-like compounds, including HemBCD (AAM04022, AAM04026, and AAM06407, respectively, from M. acetivorans and AAM02763 [HemD] from Methanopyrus kandleri AV19), catalyzing uroporphyrinogen III synthesis from the universal tetrapyrrole precursor molecule aminolevulinic acid (ALA), and SirA/CobA (AAM06406), encoding *S*-adenosyl-l-methionine-dependent uroporphyrinogen III methylase for the formation of precorrin-2 (118), were compiled. Homologs of SirC (YP_003708070 from M. voltae and AAM02708 from M. kandleri), also called precorrin-2 dehydrogenase/siroheme synthase, were identified using the homologs from Methanococcus voltae A3 (NCBI Taxonomy ID 456320) and Methanopyrus kandleri (NCBI Taxonomy ID 190192), respectively, as queries. Homologs of metal chelatase enzymes that insert iron into the sirohydrochlorin ring to make siroheme (SirB/CbiX) were not compiled in this study, since little primary sequence homology exists among characterized bacterial and archaeal homologs, despite their structural similarities (119). Homologs of proteins that store mineralized [Fe-S] clusters (IssA) were identified using the protein (Q8TZG9) from Pyrococcus furiosus strain ATCC 43587 (NCBI Taxonomy ID 186497) as the query (104) and compared to homologs with domains designated as NifB (AAM07541 from *M. acetivorans*) and NafY (PDB ID 1P90 [120] from Azotobacter vinelandii). Homologs of ferritins found in *Bacteria* were searched and compiled using the heme-dependent protein sequence of bacterioferritin (Bfr; WP_000675504) and the non-heme-dependent protein sequence of ferritin (FtnA; WP_000917208) from Escherichia coli O6:H1 as queries.

A series of criteria were used to accept sequence search results as likely homologs to the query sequences. Outputs from BLASTp queries were initially screened using an E value cutoff of 1.0e−5, which allowed identification of partial proteins in draft genomes. The sequences retrieved were filtered according to bit score, and conservative cutoffs of <30% amino acid identity and >30% query coverage were used to define homologous proteins. To minimize the chance of missing distantly related homologs, sequences with 20% to 30% identity were aligned to Clusters of Orthologous Groups (COG) (121) and Pfam (122) databases for each individual target protein and were included if they matched the COG or Pfam functional groupings. Sequences were aligned in Ugene (123) with Clustal Omega (version 1.2.4) (110). Selected alignments were further evaluated for motifs or conserved residues for each protein subunit as described in Data Set S4. Gene synteny was used to increase the confidence of *ahb* (75) and *cyd* (124) assignments when only partial sequences were identified in incomplete genomes, as these genes form parts of well-conserved operons. Sequences for IssA protein hits were aligned to proteins identified by the InterPro (125) protein family classifications, including those for the closely related NifB (IPR005980), NifX (IPR013480), and NafY (IPR031763) families as described in Vaccaro et al. (104). IssA/NifB/NifX/NafY homologs downloaded from the IPR003731 domain were combined with IssA/NafY/NifB queries and subjected to alignment with Clustal Omega (version 1.2.4) (110); the alignment was trimmed to include only the common NifX domain and remove columns with >99.78% gaps (following the methods of Vaccaro et al. [104]) and was subjected to maximum-likelihood phylogenetic analysis in IQ-TREE as described above after identifying the optimal substitution model (the LG model again, plus an R6 rate distribution). The phylogeny was compared with a recently published phylogeny (104) to demarcate functional clades from the BLASTp searches (Fig. S2).

The distribution of homologs of the subunits of the Suf [Fe-S] biosynthesis pathway was determined with BLASTp and reference sequences from E. coli strain BW2952 (NCBI Taxonomy ID 595496) and M. acetivorans, specifying an E value cutoff of 10e−5 (Data Set S3). Searches included queries of subunits SufB (WP_000089364), SufC (WP_000948863), SufD (WP_000907979), SufS (WP_000577988), SufU (WP_000331707), SufE (WP_001196530), and SufA (WP_000367160) from E. coli or SufB (AAM04369), SufC (AAM04370), and SufS (AAM04247) from M. acetivorans. The protein homologs retrieved were subjected to multiple-sequence alignment via Clustal Omega (version 1.2.4) (110) in Ugene (123). Alignments were evaluated for motifs or conserved residues for each protein subunit as listed in Data Set S4 (126). Gene synteny was used to evaluate the presence/absence of Suf homologs to increase the confidence of assignments when only partial sequences were identified in incomplete genomes (23). JGI gene identifiers for Suf protein homologs are reported in Data Set S3.

### Genomic distribution of cofactor binding motifs in inferred proteomes.

Several parallel approaches were used to identify strains potentially capable of using (siro)heme (including apparent heme auxotrophs). First, genomes were screened for proteins that contain the canonical, covalent binding motifs for tethering hemes to proteins via their vinyl side chains (designated cytochrome *c* or heme *c*), particularly in electron transfer proteins (127). Briefly, a custom Python script (128) was used to parse predicted proteins in genomes for the exact matches of the amino acid sequence motif (e.g., CXXCH, the most common motif) of interest. Genomes were also screened for other putative covalent heme binding motifs (e.g., CPV [129, 130], CX_15_CH [131], and CXXCK [132]) (Data Set S4), using the same script (128). Of the proteins identified with CX_15_CH and CXXCK motifs, all were found to include an additional CXXCH motif or were determined to likely be capable of noncovalently binding unmodified heme using methods described below. All of the proteins that encoded the CPV motif were identified as heme exporter protein subunit B (CcmB), a component of the heme modification pathway (Fig. 1) (133). Proteins with putative heme *c* binding motifs were then subjected to reciprocal BLASTp analysis against the nonredundant (NR) database and Conserved Domains Database (CDD) (134–136) to add further support to the inferred functionality of these homologs. To ensure the heme *c* binding motifs were present in proteins, alignments were conducted with the Constraint-Based Multiple-Alignment Tool (COBALT) (137) and against each other using Clustal Omega (version 1.2.4) (110).

Genomes were screened for homologs of proteins that noncovalently bind unmodified heme (designated protoheme, heme *b*, or cytochrome *b*) using a custom hidden Markov model (HMM). The HMM was constructed with HMMER version 3.3.1 (138) using homologs of protein sequences that have been experimentally confirmed to bind heme *b* in methanogens (Data Set S2). This includes the C subunit of the cytochrome *b*-containing and membrane-bound [NiFe] hydrogenase subunit (VhoC) (139) and the cytochrome *b*-containing subunit of heterodisulfide reductase (HdrE) (140). The efficacy of the HMM in capturing homologs of heme *b* binding proteins was determined by comparing the proteins retrieved using the HMM and those that were used to construct the model (e.g., VhoC [WP_048108821.1] and HdrE [AKB82465.1] in M. barkeri [NZ_CP009528.1]). Furthermore, compiled homologs were examined for the GX(H/R)XCXGX heme *b* motif (141) using the previously mentioned custom Python script. The retrieved sequences were then subjected to reciprocal BLASTp analysis against the NR database and CDD to annotate the retrieved proteins. To further validate the results, the retrieved proteins were aligned against the NCBI NR hits using COBALT (137), which allows the identification of which specific motifs are in alignment, providing more significant verification than a BLASTp score alone. The retrieved proteins were aligned against each other using Clustal Omega (version 1.2.4) in Ugene (123).

To further assess siroheme usage in the species examined here, genomes were first screened for homologs of proteins that have been biochemically shown to bind siroheme (142, 143), and then, those proteins were examined for motifs and/or biophysical characteristics suggestive of siroheme binding (142, 143). Protein queries included the dissimilatory sulfite reductase (DsrAB) from Archaeoglobus fulgidus (WP_010877930; NCBI Taxonomy ID 224325), nitrite reductase (NasD) from Haloferax mediterranei (WP_004059343; NCBI Taxonomy ID 523841), assimilatory sulfite reductase (aSir) from Methanosarcina mazei Go1 (AAM30056; NCBI Taxonomy ID 192952), and F_420_-dependent sulfite reductase (Fsr) from Methanocaldococcus jannaschii (WP_010870385; NCBI Taxonomy ID 243232), specifying a BLASTp E value cutoff of 10e−30, query coverage cutoff of 50%, and percent identification cutoff of 50% (135). Siroheme destinations are shown here as small ferredoxin-like sulfite and nitrite reductases (DsrAB, NasD, and aSir) or F_420_-like-dependent sulfite reductases (Fsr). While Fsr from Methanocaldococcus jannaschii has not been confirmed to bind heme, it possesses the siroheme binding motif and the purified protein exhibits UV-Vis absorption spectra typical of siroheme binding sulfite reductases from several taxa (144, 145), including from Desulfovibrio vulgaris, which has been demonstrated *in vitro* to bind siroheme (146). The retrieved sequences were examined for the canonical siroheme binding motifs CX_4_CX*_n_*CX_3_C and CX_5_CX*_n_*CX_3_C using the previously mentioned custom python script. The retrieved sequences were then subjected to reciprocal BLASTp analysis against the NR and CDD to add support to functional annotations (135, 136). The retrieved proteins were then aligned against each other and against the previously mentioned siroheme binding protein queries using Clustal Omega (version 1.2.4) in Ugene (123) to ensure they possessed the siroheme binding motifs.

To estimate the relative dependence of different taxa on [Fe-S] cluster proteins, the total number of proteins possessing well-described [Fe-S] cluster binding motifs or domains in each genome was quantified using MetalPredator (63), a web-based tool that applies a library of 2,390 HMMs from Pfam and the Metal Protein Database (Metal PDB) (147) to simultaneously probe for known iron-sulfur binding motifs/domains in proteins (147). All proteins retrieved were subjected to multiple sequence alignment with Clustal Omega (version 1.2.4) in Ugene (123) to verify the presence of conserved cysteine residues and putative iron-sulfur binding motifs/domains. The relative abundance of proteins predicted to bind an [Fe-S] cluster in a given genome was calculated by summing the total number of protein-coding genes that were predicted to bind an [Fe-S] cluster and dividing that by the total number of protein coding genes in that genome.

To identify patterns in Fe/S usage and deployment among genomes, a presence/absence table was generated for all of the proteins searched in the current study for each of the 326 genomes considered (Data Set S3). This table was used to calculate a Jaccard dissimilarity index using the vegan 2.5-4 package for R that was then subjected to PCO using the pco function in the labdsv 1.8-0 package for R. The percentage of variation that was explained by each axis of the ordination was calculated from the relative contributions of PCO Eigen values.
