# Supplementary material for: Pathways of Iron and Sulfur Acquisition, Cofactor Assembly, Destination, and Storage in Diverse Archaeal Methanogens and Alkanotrophs
Source: J Bacteriol. 2021 Aug 9;203(17):e00117-21. doi: 10.1128/JB.00117-21 (PMC8351635; doi:10.1128/JB.00117-21)
Supplement: Supplemental file 5 — Fig. S1 and S2. Download JB.00117-21-s0001.pdf, PDF file, 0.7 MB [file jb.00117-21-s0001.pdf]

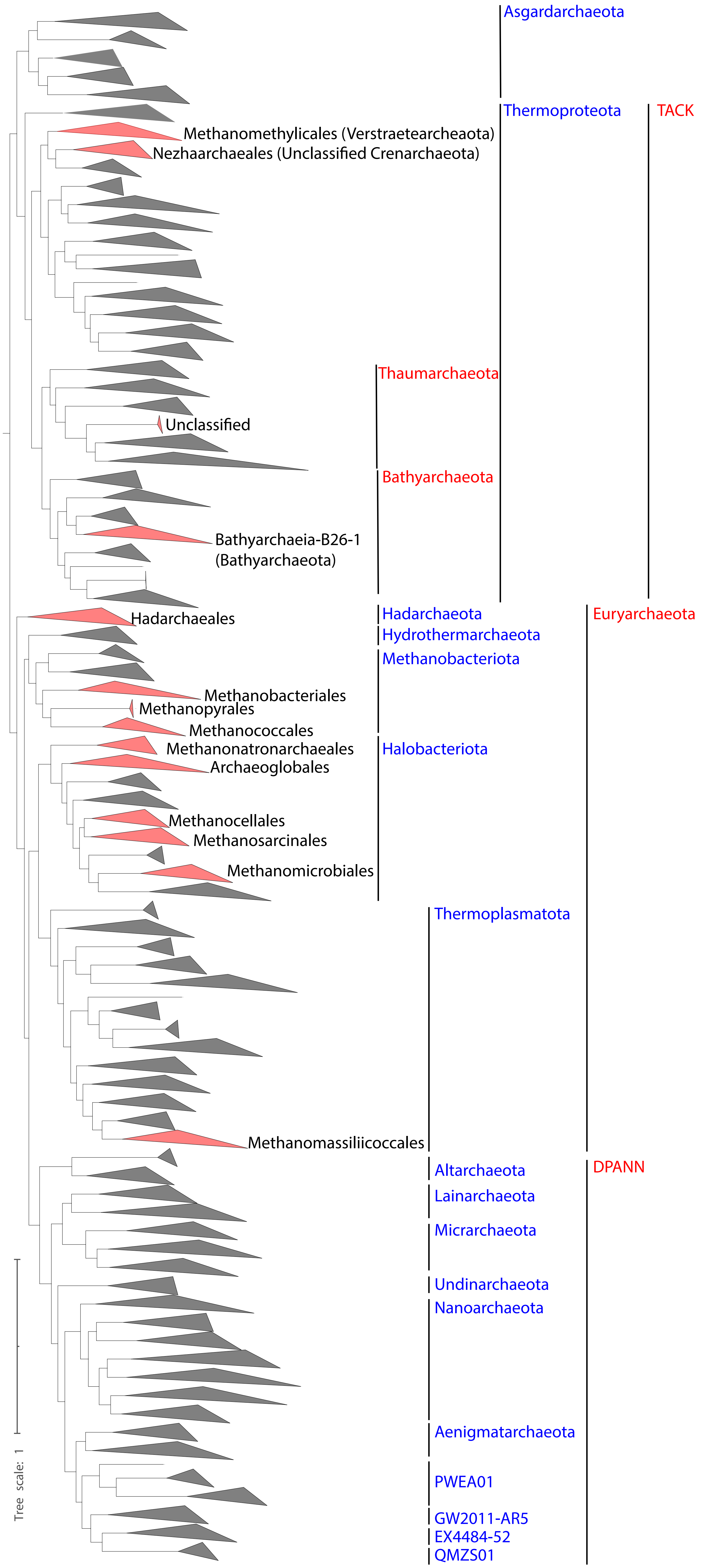

Asgardarchaeota
Thermoproteota
TACK
Methanomethylicales (Verstraetearcheaota)
Nezhaarchaeales (Unclassified Crenarchaeota)
Thaumarchaeota
Unclassified
Bathyarchaeota
Bathyarchaeia-B26-1
(Bathyarchaeota)
Hadarchaeales
Hadarchaeota
Euryarchaeota
Hydrothermarchaeota
Methanobacteriota
Methanobacteriales
Methanopyrales
Methanococcales
Methanonatronarchaeales
Halobacteriota
Archaeoglobales
Methanocellales
Methanosarcinales
Methanomicrobiales
Thermoplasmatota
Methanomassiliicoccales
Altarchaeota
DPANN
Lainarchaeota
Micrarchaeota
Undinarchaeota
Nanoarchaeota
Aenigmatarchaeota
PWEA01
GW2011-AR5
EX4484-52
QMZS01
Tree scale: 1

Fig. S1. Maximum Likelihood phylogenomic analysis of 2,337 archaeal genomes from the Genome Taxonomy Database (GTDB). A multi-sequence alignment for the genomes was retrieved from the GTDB dataset and comprised 122 single-copy housekeeping genes and 5,125 alignment positions. Curated taxonomic information from the database was matched to the alignments and the order-level lineages are collapsed as triangles within the tree. Orders that are represented in the 326 genome dataset used in this study are highlighted in red and the GTDB taxonomic classification name is given next to the clade. If the GTDB order-level classification differs from the taxonomic name used in the manuscript, the latter is shown in parentheses. Widely recognized higher-order groups (e.g., phyla) that have been previously established are shown in red, while phylum-level groups established within the GTDB database classification scheme are shown in blue, for reference. Scale at the bottom left shows the expected number of substitutions per site in the alignment.

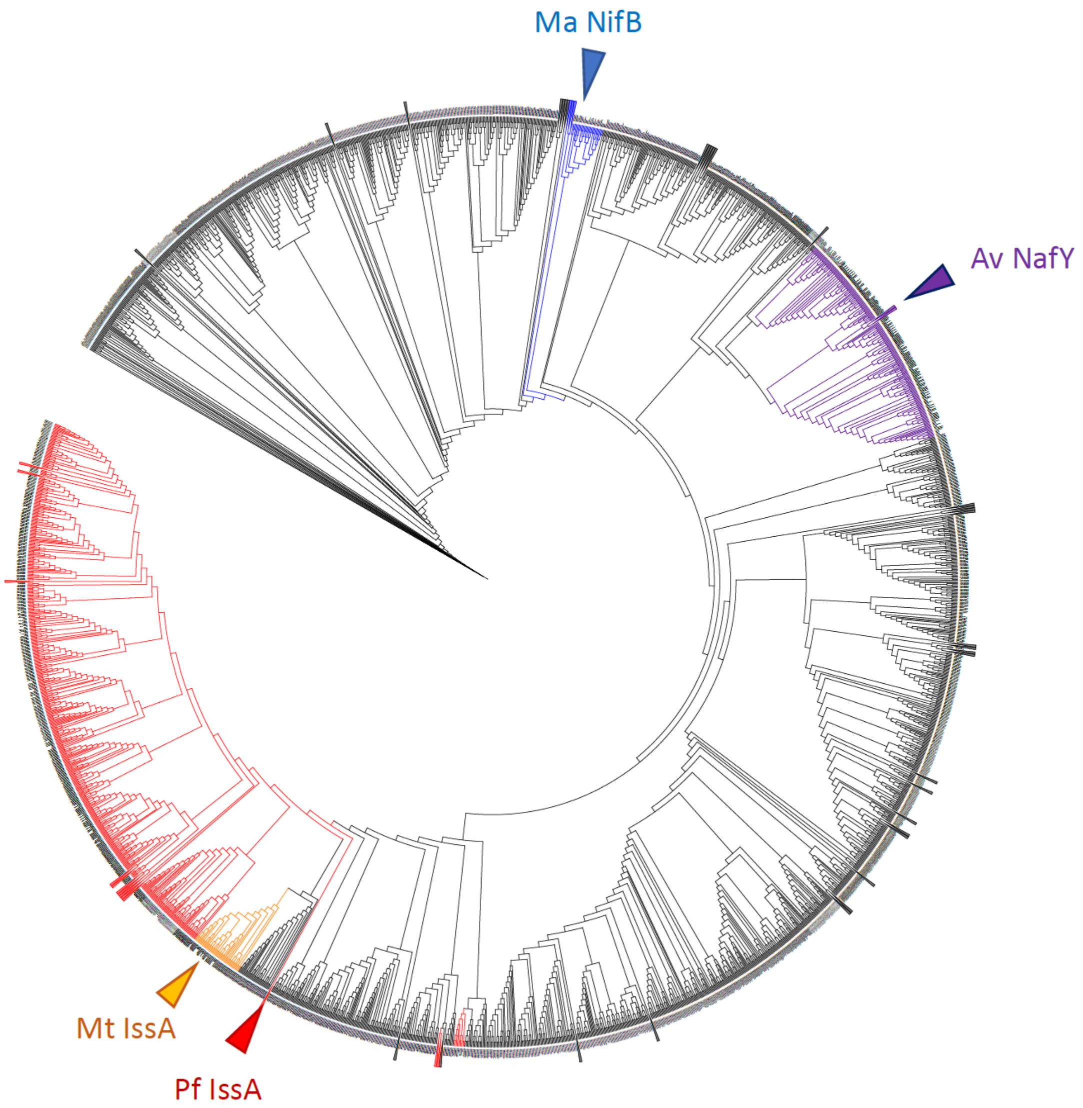

Fig. S2. Maximum-Likelihood phylogenetic reconstruction of IPR003731 domain containing proteins including homologs of NifB, NafY and IssA. Colored clades contain members of the NifB (blue, *Methanosarcina acetivorans* (AAM07541)), NafY (purple, *Azotobacter vinelandii* (Q9F5X9)) and IssA protein families (*Pyrococcus furiosus* (Q8TZG9) and *Methanobacterium thermoautotrophicum* (Q72A89)). Reference sequences are shown by colored arrowheads corresponding to the coordinated reference protein. Those lineages that are derived from and that cluster with IssA from *P. furiosus* and *M. thermoautotropicum* are colored red and were used to demarcate IssA homologs in the archaeal methanogen and alkanotroph genomic dataset analyzed herein. The phylogeny was generated in IQ-TREE after identifying the optimal substitution model (again the LG model + R6 rate distribution) with an alignment block that was trimmed to include shared residues and to remove columns with >99.78% gaps (to 84 amino acids).
